# The constrained brain in multiple sclerosis: Cognitive impairment is related to network-specific coupling of structural and functional connectivity

**DOI:** 10.1177/20552173261448282

**Published:** 2026-06-05

**Authors:** Marloes DA Bet, Maureen van Dam, Tommy AA Broeders, Aurélie Ruet, Frederik Barkhof, Eva MM Strijbis, Linda Douw, Ismail Koubiyr, Hanneke E Hulst, Menno M Schoonheim

**Affiliations:** MS Center Amsterdam, Anatomy & Neurosciences, Vrije Universiteit Amsterdam, 601873 Amsterdam Neuroscience, 1209 Amsterdam UMC location VUmc, Amsterdam, the Netherlands; MS Center Amsterdam, Anatomy & Neurosciences, Vrije Universiteit Amsterdam, 601873 Amsterdam Neuroscience, 1209 Amsterdam UMC location VUmc, Amsterdam, the Netherlands; Health, Medical and Neuropsychology Unit, and Leiden Institute of Brain and Cognition (LIBC), Leiden University, Institute of Psychology, Leiden, the Netherlands; MS Center Amsterdam, Anatomy & Neurosciences, Vrije Universiteit Amsterdam, 601873 Amsterdam Neuroscience, 1209 Amsterdam UMC location VUmc, Amsterdam, The Netherlands; Neurocentre Magendie, Université de Bordeaux, Bordeaux, France; Service de neurologie CHU de Bordeaux, Université de Bordeaux, Bordeaux, France; MS Center Amsterdam, Radiology and Nuclear Medicine, Vrije Universiteit Amsterdam, 601873 Amsterdam Neuroscience, 1209 Amsterdam UMC location VUmc, Amsterdam, the Netherlands; Queen Square Institute of Neurology and Centre for Medical Image Computing, 4919 University College London, London, UK; MS Center Amsterdam, Neurology, Vrije Universiteit Amsterdam, 601873 Amsterdam Neuroscience, 1209 Amsterdam UMC location VUmc, Amsterdam, the Netherlands; Department of Anatomy and Neurosciences, 601873 Amsterdam Neuroscience, Cancer Center Amsterdam, Amsterdam University Medical Centers location Vrije Universiteit Amsterdam, Amsterdam, the Netherlands; Systems & Network Neuroscience, 601873 Amsterdam Neuroscience, Amsterdam, the Netherlands; MS Center Amsterdam, Anatomy & Neurosciences, Vrije Universiteit Amsterdam, 601873 Amsterdam Neuroscience, 1209 Amsterdam UMC location VUmc, Amsterdam, the Netherlands; Health, Medical and Neuropsychology Unit, and Leiden Institute of Brain and Cognition (LIBC), Leiden University, Institute of Psychology, Leiden, the Netherlands; MS Center Amsterdam, Anatomy & Neurosciences, Vrije Universiteit Amsterdam, 601873 Amsterdam Neuroscience, 1209 Amsterdam UMC location VUmc, Amsterdam, The Netherlands

**Keywords:** Structure-function coupling, multiple sclerosis, magnetic resonance imaging, cognition, connectivity

## Abstract

**Background:**

Cognitive impairment in multiple sclerosis (MS) is related to abnormal structural and functional brain network changes. As both modalities have mostly been studied separately, structure connectivity-function coupling (SC-FC) may elucidate how function is constrained by structure.

**Objectives:**

We examine how changes in SC-FC and its dynamics SC-FC (dSC-FC) may underlie MS-related cognitive impairment.

**Methods:**

We classified 266 people with MS (PwMS) as cognitively impaired (CI; *z* < −2 on ≥2/7 cognitive abilities compared to healthy controls [HC]) or preserved (CP; *z* > −1.5 on ≥2/7 abilities). Derived from diffusion-weighted and functional magnetic resonance imaging, SC-FC reflected the correlation coefficient between structural and functional connectivity, while dSC-FC reflected its variability. Whole-brain, within-network, and between-network (d)SC-FC were compared between 95 HC, 197 CP, and 69 CI.

**Results:**

Compared to CP and HC, CI showed higher SC-FC for connections extending from the dorsal attention (DAN; *p* ≤ 0.006) and somatomotor networks (*p* ≤ 0.024), and higher dSC-FC within the visual network (*p* ≤ 0.003) and deep grey matter (*p* ≤ 0.006). SC-FC in connections extending from the DAN predicted CP/CI status in PwMS (*β* = 0.069, OR = 1.072 [95% CI = 1.009–1.139], *p* = 0.025).

**Conclusions:**

In MS-related CI, deterioration of structural pathways is paired with loss of functional strength in connections extending from the DAN. This may hinder DAN-mediated stimulus selection, possibly contributing to CI.

## Introduction

Cognitive impairment (CI) occurs in up to 65% of people with multiple sclerosis (PwMS)^1^ and is associated with brain network features of both structure (disrupted anatomical white matter (WM) tracts) and function (abnormal connectivity patterns).^2^ Although both structural connectivity (SC) and functional connectivity (FC) alterations have been observed in multiple sclerosis (MS),^3^ they have mostly been studied separately. Since it remains unclear what drives functional changes in MS, examining structure-function interactions can offer novel conceptual and mechanistic insights into how MS pathology affects cognitive performance.^4^

Structure connectivity-function coupling (SC-FC) represents the correlation coefficient between SC and FC. Conceptually, SC-FC captures the extent of correspondence of structural and functional network strength across network edges, such that simultaneously high SC and FC yields high SC-FC values, but damaged structural connections accompanied by unchanged FC strength yield low SC-FC values. In healthy subjects, SC-FC shows within-subject reproducibility, varies across brain regions and networks, and correlates with demographics and behavioral outcomes.^5^ Further, SC-FC is higher in visual and subcortical regions, but lower in regions involved in more higher-order cognitive functions.^5^

In clinically isolated syndrome (CIS), SC-FC is lower compared to healthy controls (HC), yet over time, increasing SC-FC is associated with worsening cognition,^6,7^ although this has not been studied in later disease stages. Given the gradually deteriorating structural connections in MS, we expect higher overlap of structure and function in cognitive networks, representing a diminished capacity for spatial diversity of FC along anatomical tracts, which impairs cognition.^4^ Specifically, we expect higher SC-FC in resting-state networks^8^ involved in cognitive processing, like the default-mode network (DMN), involved in conceptual and self-processing^9^; the frontoparietal network (FPN), involved in modulating cognitive control and goal-directed behavior;^10^ and the dorsal attention network (DAN), involved in directing attention toward goal-relevant stimuli.^11^

While FC is conventionally averaged across the BOLD signal, cognition relies on dynamic (time-varying) fluctuations of interactions between resting-state networks (RSNs) in the brain.^12^ Thus, some flexibility in SC-FC is needed for the functional network to dynamically use the structural infrastructure to efficiently process information. In PwMS, FC dynamics are indeed related to cognition,^13^ but it remains unknown how this relates to the (damaged) underlying structural network. Mapping the variability of SC-FC (dSC-FC) may therefore show whether structural damage is paired with altered FC variability in PwMS. We postulate that higher damage-induced structural constraints on function limit the brain's capacity for time-varying reconfiguration, and thereby contribute to cognitive impairment. By calculating the variability of structure-function interactions over time, beyond consistent disruptions of this interaction (i.e. continually low FC), we aimed to capture time-varying abnormalities in this interaction (i.e. more or less extreme FC peaks) that less directly impact the overall SC-FC value. In healthy subjects, SC-FC variability is region-specific and related to the distribution of anatomical connection lengths.^14^

Here, we investigated network-level interactions between structure and function in PwMS.^8^ Because structural connectivity (SC) is typically lower in PwMS than in HC, we interpreted SC-FC as the extent to which function is constrained by (gradually deteriorating) structural pathways. Higher-than-normal SC-FC indicated that FC, too, had decreased; and lower-than-normal SC-FC indicated that, despite structural damage, FC between directly connected regions could remain relatively high. We hypothesized that, especially in the DMN, FPN, and DAN, cognitively impaired (CI) PwMS would show higher SC-FC and lower dSC-FC than cognitively preserved PwMS.

## Materials and methods

### Study procedures

**Figure 1.**
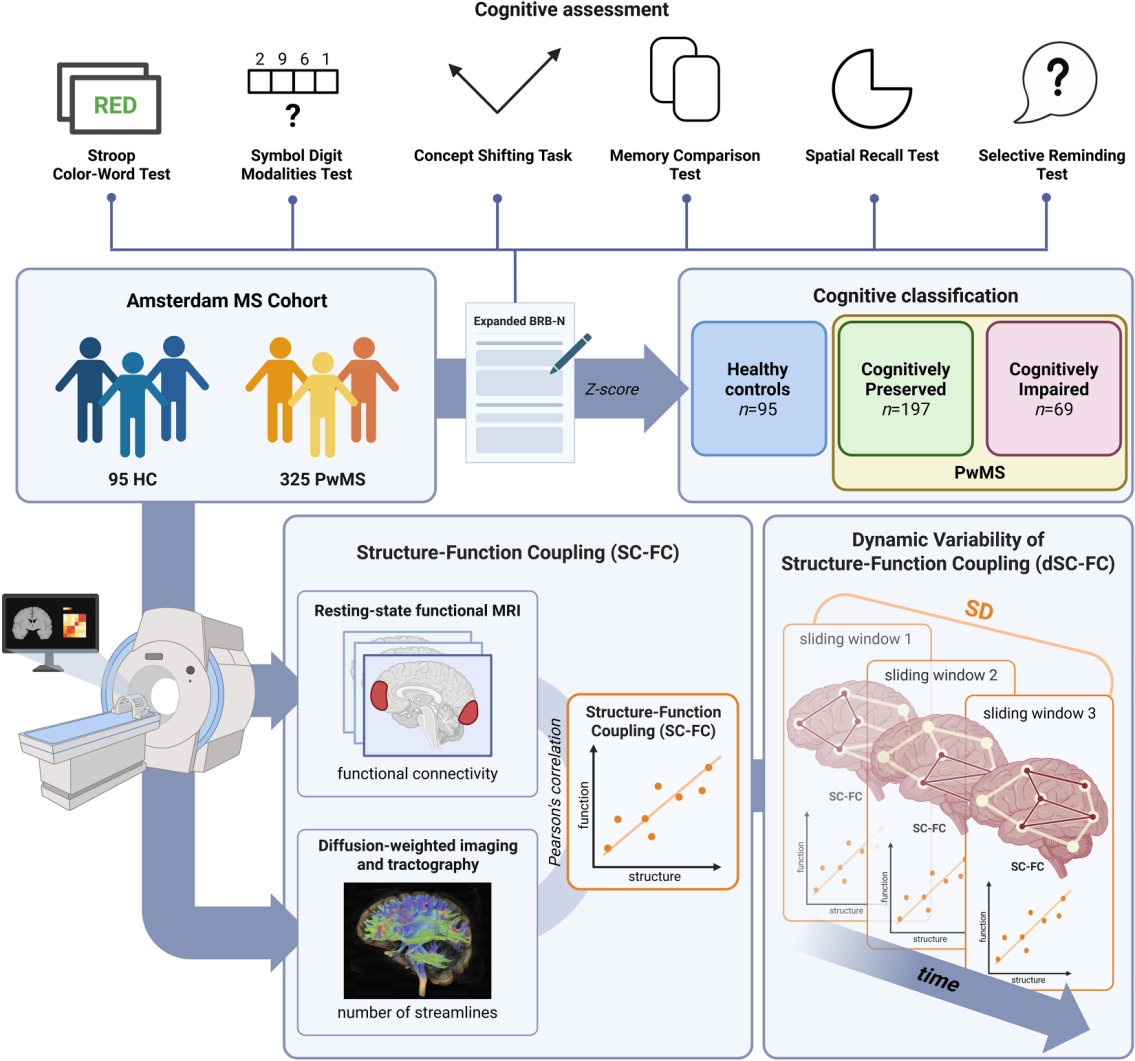
A subsample of the Amsterdam MS cohort, consisting of both PwMS and HC, underwent cognitive testing using an expanded Dutch version of the BRB-N. Test scores were *z*-transformed based on normative data of the HC sample; accordingly, PwMS were classified as cognitively preserved or cognitively impaired. All participants underwent magnetic resonance imaging, yielding a resting-state functional image, from which we calculated functional connectivity per brain region, and a diffusion-weighted image, to which tractography was applied to obtain the number of streamlines between brain regions. The resulting structural and functional connectivity matrices were correlated, and Pearson's *r* herein reflected SC-FC, or the overlap of the structural and functional networks in those connections. Further, we split the functional time-series up into overlapping sliding windows and calculated a functional connectivity matrix for each time window. Within each sliding window, we again correlated the structural connectivity matrix to the time-varying functional connectivity matrix. From these SC-FC values per window over time, we calculated the standard deviation per person to reflect the dynamic variability of SC-FC (dSC-FC). MS: multiple sclerosis; HC: healthy controls; PwMS: people with multiple sclerosis; BRB-N: Brief Repeatable Battery (Dutch version); SC-FC: structure connectivity-function coupling; MRI: magnetic resonance imaging; dSC-FC: dynamic structure connectivity-function coupling; SD: standard deviation*.* Created with BioRender.com.

We included 95 HC and 324 PwMS, who were diagnosed according to McDonald criteria^15^ and relapse- and steroid-free for at least two months, from the Amsterdam MS cohort.^13^ Education level was estimated using the Verhage classification and dichotomized such that scores >5 reflected higher education.^16^ Physical disability was assessed by a certified examiner using the Expanded Disability Status Scale (EDSS).^17^ Participants underwent magnetic resonance imaging (MRI) and neuropsychological testing (Figure 1).

### Neuropsychological examination

An expanded Dutch Brief Repeatable Battery of Neuropsychological Tests was administered to measure cognitive abilities as described in the Diagnostic and Statistical Manual of Mental Disorders (5th edition). The Symbol Digit Modalities Test and the Stroop Color-Word Test, respectively, assessed information processing speed and behavioral inhibition, as part of the domain of complex attention; the Concept Shifting Task and the Memory Comparison Test respectively assessed mental flexibility and working memory, as part of executive function; and the Selective Reminding Test and the Spatial Recall Test respectively assessed free recall of recent verbal and spatial information, as part of the learning and memory domain. All cognitive test scores were *z*-transformed based on HC and corrected for age, sex, and education level.

PwMS were classified as CI if they scored *z* < −2.0 SD on two or more cognitive abilities, and as mildly CI (MCI) if they scored *z* < −1.5 SD compared to HC on two or more cognitive abilities. Otherwise, PwMS were classified as cognitively preserved (CP).^18^ To enhance contrast between groups and to limit the number of statistical tests, MCI (*n* = 58) were not considered further.

### Magnetic resonance imaging

#### Image acquisition protocol

MR images were acquired using a 3-Tesla whole-body scanner (General Electric Signa-HDxt, Milwaukee, WI, USA) with an eight-channel head coil. The protocol included a 3D-T1 weighted fast spoiled gradient echo sequence (repetition time (TR) = 7.8 ms, echo time (TE) = 3.22 ms, inversion time (TI) = 450 ms, flip angle = 12°, 1.0 mm sagittal slices, and 0.94 × 0.94 mm^2^ in-plane resolution), a 3D fluid-attenuated inversion recovery (FLAIR) sequence (TR = 8000 ms, TE = 125 ms, TI = 2350 ms, 1.2 mm sagittal slices, and 0.98 × 0.98 mm^2^ in plane resolution), diffusion-weighted imaging (DWI, 30 directions, five volumes with *b* = 0 s/mm^2^ and 30 volumes with *b *= 1000 s/mm^2^, TE = 91 ms, TR = 13 ms, flip angle = 90°, 2.4 mm axial slices, and 2 × 2 mm^2^ in-plane resolution), and resting-state functional MRI with 202 volumes, of which the first two were discarded (echo-planar imaging, TR = 2200 ms, TE = 35 ms, flip angle = 20°, 3 mm axial slices, and 3.3 × 3.3 mm^2^ in-plane resolution).

#### Segmentation of lesions and tissue volumes

WM lesion load was quantified using *k*-nearest neighbor interpolation with tissue-type priors on FLAIR and T1-weighted images.^19^ From lesion-filled images, grey matter (GM) and WM volumes were calculated using SienaX for cortical regions and FIRST for deep GM regions (FSL-v5),^20^ and corrected for intracranial volume.

#### Functional preprocessing

Resting-state functional MR images were preprocessed using a custom FSL-FEAT-based pipeline, available at https://github.com/marloesbet/SC-FC. After brain extraction, motion correction, and 5 mm spatial smoothing, we linearly registered functional images to T1-space, applied ICA-AROMA to remove physiological noise, regressed out average WM and CSF signal, and applied a high-pass filter. We created a mask to exclude significantly distorted voxels based on the robust intensity range.^21^ After correlating time series, FC matrices were normalized using Fisher's *r*-to-*z* transformation and made absolute.

#### Diffusion preprocessing

Diffusion-weighted images were preprocessed using QSIPrep (part of FreeSurfer-v6).^22^ To perform probabilistic Anatomically Constrained Tractography (ACT) in MRtrix-v3,^23^ we generated a segmented tissue image using 5ttgen, which was registered to T1-space using Advanced Normalization Tools (ANTs). A single-shell response function estimated fiber orientation distributions per voxel based on constrained spherical deconvolution,^24^ and 10 million streamlines were generated through random seeding. To prevent disproportional weighting of large tracts and improve biological plausibility, streamlines were SIFT-corrected, yielding one million streamlines in total.^25^ We chose tract count over fractional anisotropy as our SC metric to ensure that the weights of the structural connections reflected connectivity strength, analogous to our methodology concerning FC.

#### Regional parcellation

The Brainnetome Atlas was registered to T1-space and overlaid on the SienaX-derived segmentation to delineate cortical GM areas, which were combined with FIRST-derived subcortical areas to form a composite atlas containing 210 cortical and 14 subcortical regions (cerebellum excluded). Atlases were linearly registered to diffusion and functional space using transformation matrices produced by ANTs. Based on the aforementioned distortion masks, we excluded regions with ≤30% voxel coverage in ≥90% of subjects in either the structural or functional data.^21^ The 30 excluded regions primarily encompassed limbic regions and the nucleus accumbens (Supplemental Table S1). Using maximal overlap, the remaining 194 regions were grouped into RSNs: default-mode (DMN), frontoparietal (FPN), dorsal attention (DAN), ventral attention (VAN), somatomotor (SMN), visual (VN), and deep GM (DGM) networks.^8^

#### Structure-function coupling

**Figure 2.**
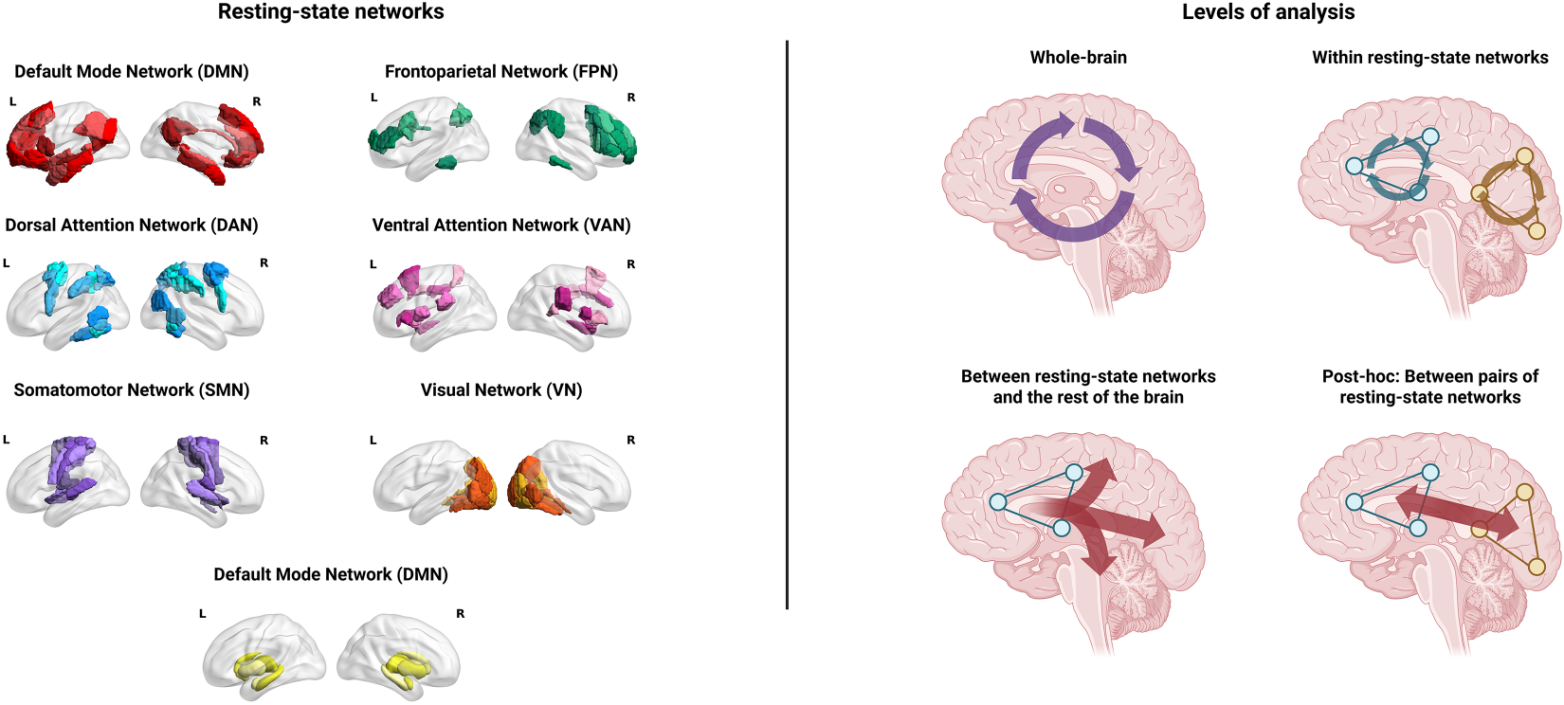
Besides whole-brain analyses, we conducted analyses per resting-state network (RSN) based on Yeo et al., (2011)^8^, visualized on the left. Structural and functional connectivity were calculated for all direct connections across the whole brain (top left of the figure on the right); direct connections within each resting-state network (top right of the figure on the right); direct connections between each RSN and all other regions of the brain (bottom left of the figure on the right); and, in case of significant effects in tracts between an RSN and the rest of the brain, a post-hoc analysis of direct connections between the RSN in question and all other RSNs (bottom right of the figure on the right). Created with BioRender.com.

Zeros in the SC matrix were excluded to retain only direct connections. The structural vector containing the remaining streamline counts per region was reordered from region index order (1 through 194) and assigned to a value in a Gaussian distribution.^6^ SC-FC was operationalized as the Pearson correlation coefficient between the SC and FC vectors. We calculated SC-FC for all connections in the brain; connections within each of the RSNs; and connections between each of the RSNs and the rest of the brain (Figure 2).

**Figure 3.**
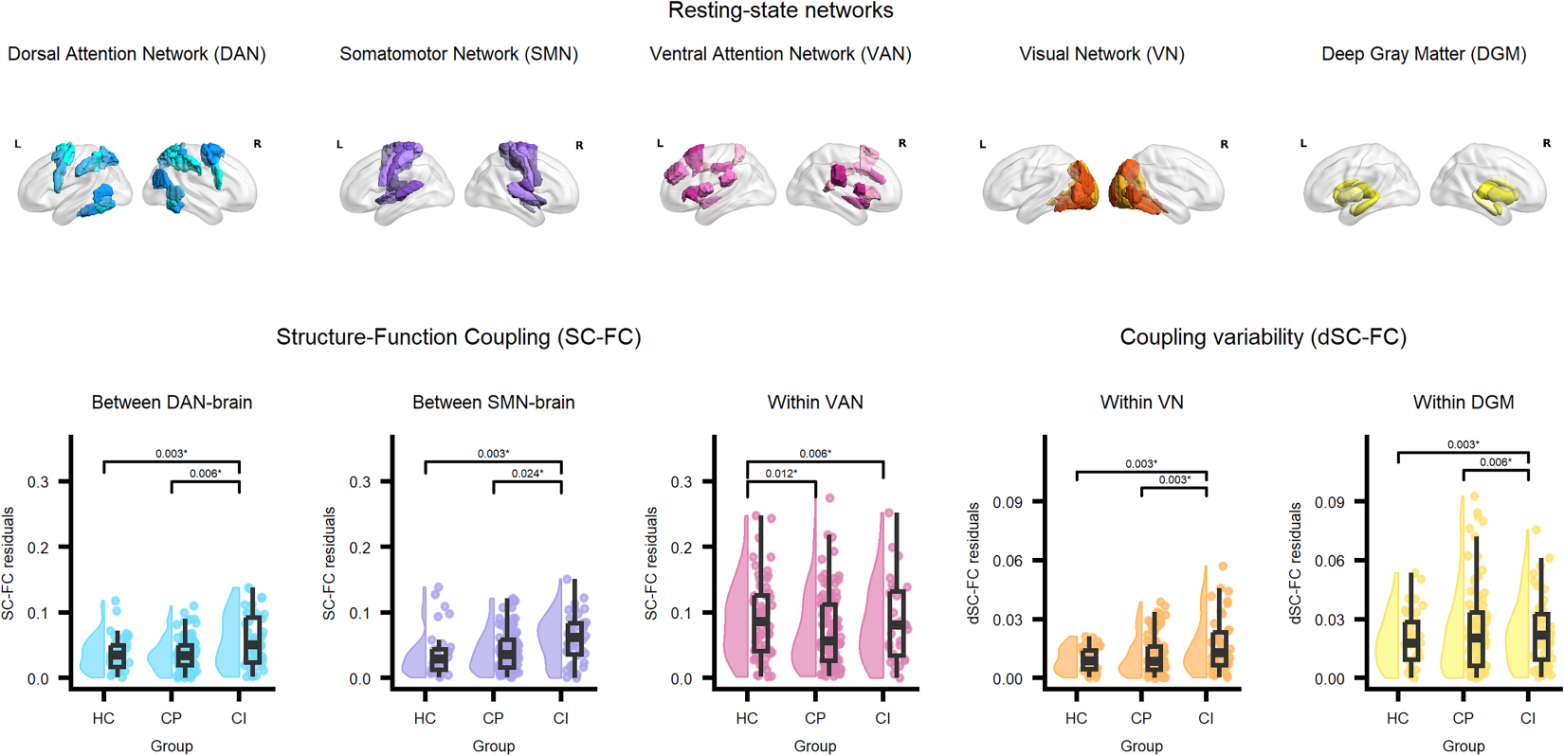
Values on the *y*-axis represent residuals of outcome variables after correction for sex, age, and education level. Reported *p*-values correspond to contrast tests and are Bonferroni-corrected. HC: healthy controls; PwMS: people with multiple sclerosis; CP: cognitively preserved; CI: cognitively impaired.

#### SC-FC variability

We calculated coupling variability (dSC-FC) using sliding windows, for which time series were split into 174 overlapping windows containing 27 volumes, with step-size one.^26^ After calculating SC-FC for each window, we calculated the SD of SC-FC across all windows, resulting in dSC-FC, a single-value outcome proxy measure for network flexibility, reflecting the temporal variability of SC-FC.

### Statistical analyses

All (d)SC-FC comparisons were performed using ANCOVA with group (HC/CP/CI) as predictor and sex, age, and education level (dichotomized) as covariates. Applying Bonferroni correction for the number of RSNs and the number of coupling variables evaluated, *p*-values below *α* = 0.0035 were considered statistically significant. Pairwise contrasts between HC, CP, and CI were also Bonferroni-corrected.

Using a hierarchical testing strategy, we conducted post-hoc analyses for (d)SC-FC in RSN-RSN connections only in case of significant group differences in between-RSN (d)SC-FC. Again, we applied the Bonferroni correction for the number of RSNs.

#### Logistic regression of cognitive status

Using backward elimination in a logistic regression model, we examined whether SC-FC variables showing group differences were predictive of cognitive status (CP[*n* = 197]/CI[*n* = 69]) beyond demographics and measures of structural damage. Values for GM volume and lesion volume were rescaled to deciliters and milliliters, respectively, to improve the interpretability of odds ratios. In case of multicollinearity of two predictors (*r* > 0.50), the least significant predictor was excluded. To evaluate the robustness of the model, we performed bootstrapping with replacement of cases with 5000 iterations, yielding 95% confidence intervals for the beta coefficients in the model. From the precision and recall of the model, we calculated the F1-value to quantify model performance using the following formulas:

$$\mathrm{Precision}=\frac{\mathrm{True}\;\mathrm{Positives}}{\mathrm{True}\;\mathrm{Positives}+\mathrm{False}\;\mathrm{Positives}}$$

$$\mathrm{Recall}=\frac{\mathrm{True}\;\mathrm{Positives}}{\mathrm{True}\;\mathrm{Positives}+\mathrm{False}\;\mathrm{Negatives}}$$

$$F1=2*\frac{\mathrm{Precision}*\mathrm{Recall}}{\mathrm{Precision}+\mathrm{Recall}}$$

### Sensitivity analyses

For the (d)SC-FC variables showing significant CP/CI differences, we explored whether these predicted individual cognitive abilities using linear regression with (d)SC-FC as the predictor and sex, age, and education level as covariates.

To verify our interpretation of SC-FC as higher overlap of SC and FC due to deteriorating SC, we ascertained whether the strength of SC was indeed lower in PwMS versus HC and in CI versus CP and HC, and checked the directionality of group differences in FC. Further, to investigate whether effects were independent from physical disability, we conducted analogous analyses for low vs. high disability (EDSS >4.0).^27^ For further validation, we repeated the main analyses, including the MCI group.

Additionally, we estimated the effect of grey matter volume on the estimated models for (d)SC-FC measures. Finally, we compared mean framewise displacement on DWI and fMRI scans between HC and PwMS. In case of significant differences, we corrected both our main analyses and modality-specific sensitivity analyses for mean motion.

## Results

### Demographics

Age and education level were similar between PwMS (*n* *=* 266) and HC (*n* *=* 95). Most of the PwMS (68.8%) and HC (57.9%) were women. Of PwMS, 197 (74.1%) presented with relapsing-remitting MS, and 163 (61.3%) were on disease-modifying treatment. PwMS had lower brain volumes than HC and scored worse on all cognitive abilities (all *p* < 0.001; Table 1).

**Table 1.** Overview of demographics, clinical, and cognitive functioning.

	HC (*n* = 95)	PwMS (*n* = 266)	*p*	CP (*n* = 197)	CI (*n* = 69)	*p* ^a^
**Demographics**						
Sex (women: men (*%*))	55: 40 (57.9%)	183: 83 (68.8%)	0.054	144: 53 (73.10%)	39: 30 (56.5%)	0.011*
Age	45.70 ± 10.35	48.11 ± 10.68	0.058	46.67 ± 10.51	52.20 ± 10.13	<0.001*
Education level	6.00 (4.00–7.00)	5.00 (4.00–6.00)	0.288	5.00 (4.00–6.00)	4.00 (3.00–6.00)	0.003*
**Clinical functioning**						
MS type (RRMS/SPMS/PPMS)	NA	197/46/23	NA	158/24/15	39/22/8	<0.001*
Disease duration (*years since diagnosis*)	NA	14.68 ± 8.45	NA	13.70 ± 7.98	18.10 ± 9.14	<0.001*
EDSS	NA	3.50 (2.00–5.00)	NA	3.00 (2.00–4.00)	4.50 (3.50–6.50)	<0.001*
Disease-Modifying Treatment (*yes/no/unknown*)	NA	163/77/26	NA	119/60/18	44/17/8	0.604
**Cognitive test scores (*z*-scored based on HC)**						
Stroop Color-Word Test	0.02 ± 0.63	−0.60 ± 1.20	<0.001*	−0.25 ± 0.79	−1.60 ± 1.55	<0.001*
Concept Shifting Task	0.00 ± 0.67	−0.68 ± 1.23	<0.001*	−0.23 ± 0.71	−1.99 ± 1.47	<0.001*
Symbol Digit Modalities Test	0.01 ± 1.00	−1.09 ± 1.43	<0.001*	−0.49 ± 0.99	−2.83 ± 1.04	<0.001*
Selective Reminding Test	0.01 ± 0.90	−0.41 ± 1.15	<0.001*	−0.01 ± 0.88	−1.54 ± 1.09	<0.001*
Spatial Recall Test	0.01 ± 1.00	−0.53 ± 1.25	<0.001*	−0.20 ± 1.07	1.46 ± 1.25	<0.001*
Memory Comparison Test	0.00 ± 0.85	−1.00 ± 1.56	<0.001*	−0.48 ± 0.85	−2.51 ± 2.08	<0.001*
**Normalized brain volumes**						
Total brain volume (L)	1.51 ± 0.07	1.45 ± 0.08	<0.001*	1.48 ± 0.07	1.40 ± 0.08	<0.001*
Cortical grey matter volume (ml)	0.78 ± 0.05	0.75 ± 0.05	<0.001*	0.76 ± 0.05	0.72 ± 0.05	<0.001*
Deep grey matter volume (ml)	62.95 ± 3.73	55.98 ± 7.04	<0.001*	58.18 ± 5.18	49.72 ± 7.86	<0.001*
FLAIR lesion volume (ml)	NA	13.56 (6.81–25.82)	NA	10.98 (5.99–18.17)	28.91 (17.51–45.70)	<0.001*

HC: healthy controls; PwMS: People with multiple sclerosis; CP: cognitively preserved; CI: cognitively impaired; RRMS: relapsing-remitting multiple sclerosis; SPMS: secondary progressive multiple sclerosis; PPMS: primary progressive multiple sclerosis; EDSS: Expanded Disability Status Scale; FLAIR: fluid-attenuated inversion recovery; NA: not applicable.

Displayed are the mean ± standard deviation for normally distributed continuous variables and median (interquartile range) for ordinal or non-normally distributed variables.

a Displaying significance between healthy controls, people with MS who were cognitively preserved, and people with MS who were cognitively impaired.

*Significant group difference at the *α* = 0.05 level.

Of PwMS, 197 (74.1%) were classified as CP and 69 (25.9%) as CI. PwMS with CI were ∼5.5 years older than CP PwMS (*p* < 0.001), more frequently male (*p* = 0.011), and had a lower education level (*p* = 0.003). Fewer CI PwMS were diagnosed with RRMS (*p* < 0.001). Further, CI PwMS had longer disease duration, higher EDSS, smaller brain volumes, and scored worse than CP on all cognitive abilities (all *p* < 0.001).

### SC-FC: Cognitive status

Table 2 shows group differences in SC-FC. Whole-brain SC-FC was similar between SC-FC between HC, CP, and CI (*p* = 0.114).

**Table 2.** Group comparisons of structure-function coupling.

	HC (*n* = 95)	CP (*n* = 197)	CI (*n* = 69)	*F*	*η* ^2^	*p*
**(1) Whole-brain structure-function coupling (*α* = 0.025)**						
Whole-brain	0.289 ± 0.031	0.295 ± 0.041	0.304 ± 0.068	2.181	0.012	0.114
**(2) Structure-function coupling within networks (*α* = 0.0035)**						
Default-mode network	0.366 ± 0.060	0.369 ± 0.066	0.365 ± 0.100	0.117	0.001	0.889
Frontoparietal network	0.344 ± 0.099	0.340 ± 0.101	0.358 ± 0.126	0.716	0.004	0.489
Dorsal attention network	0.293 ± 0.095	0.317 ± 0.102	0.334 ± 0.119	3.188	0.018	0.042
Ventral attention network	0.400 ± 0.100	0.361 ± 0.104	0.344 ± 0.133	6.085	0.033	0.003*^a,c^
Somatomotor network	0.360 ± 0.073	0.367 ± 0.078	0.353 ± 0.111	0.695	0.004	0.500
Visual network	0.267 ± 0.067	0.279 ± 0.082	0.298 ± 0.104	2.660	0.015	0.071
Deep gray matter	0.149 ± 0.124	0.142 ± 0.125	0.184 ± 0.166	2.494	0.014	0.084
**(3) Structure-function coupling between networks – rest of brain (*α* = 0.0035)**						
Default-mode network	0.247 ± 0.050	0.249 ± 0.052	0.260 ± 0.065	1.194	0.007	0.304
Frontoparietal network	0.199 ± 0.054	0.200 ± 0.058	0.205 ± 0.080	0.170	0.001	0.844
Dorsal attention network	0.231 ± 0.049	0.234 ± 0.051	0.259 ± 0.070	6.037	0.033	0.003*^b,c^
Ventral attention network	0.254 ± 0.047	0.248 ± 0.052	0.256 ± 0.066	0.827	0.005	0.438
Somatomotor network	0.238 ± 0.052	0.253 ± 0.057	0.276 ± 0.072	8.065	0.043	<0.001*^b,c^
Visual network	0.234 ± 0.083	0.258 ± 0.080	0.265 ± 0.086	3.708	0.020	0.025
Deep gray matter	0.128 ± 0.071	0.139 ± 0.076	0.148 ± 0.066	1.525	0.009	0.219
**(4) Structure-function coupling between pairs of networks (α = 0.0035)**						
Dorsal attention network-default-mode network	0.236 ± 0.083	0.236 ± 0.089	0.265 ± 0.103	2.740	0.015	0.066
Dorsal attention network-frontoparietal network	0.239 ± 0.086	0.221 ± 0.088	0.228 ± 0.107	1.144	0.006	0.320
Dorsal attention network-ventral attention network	0.243 ± 0.084	0.239 ± 0.087	0.250 ± 0.111	0.363	0.002	0.696
Dorsal attention network-somatomotor network	0.306 ± 0.077	0.306 ± 0.089	0.328 ± 0.107	1.599	0.009	0.204
Dorsal attention network-visual network	0.300 ± 0.099	0.311 ± 0.099	0.310 ± 0.122	0.352	0.002	0.703
Dorsal attention network-deep gray matter	0.055 ± 0.152	0.081 ± 0.156	0.076 ± 0.139	0.963	0.005	0.383
Somatomotor network-default mode network	0.263 ± 0.099	0.288 ± 0.096	0.286 ± 0.107	2.201	0.012	0.112
Somatomotor network-frontoparietal network	0.036 ± 0.105	0.033 ± 0.094	0.028 ± 0.104	0.113	0.001	0.893
Somatomotor network-ventral attention network	0.339 ± 0.067	0.342 ± 0.078	0.365 ± 0.094	2.428	0.013	0.090
Somatomotor network-visual network	−0.080 ± 0.164	−0.080 ± 0.179	−0.105 ± 0.171	0.554	0.003	0.575
Somatomotor network-deep gray matter	0.019 ± 0.148	0.029 ± 0.154	0.056 ± 0.130	1.234	0.007	0.292

HC: healthy controls; CP: cognitively preserved people with multiple sclerosis; CI: cognitively impaired people with multiple sclerosis.

Displayed are the mean ± standard deviation. Reported values are corrected for age, sex, and education level (binarized).

a Significant difference between HC and CP.

b Significant difference between CP and CI.

c Significant difference between HC and CI.

* Significant group difference at the alpha-level (*α*) reported in the subheader.

Significant group differences in network-level SC-FC are shown in Figure 3. Concerning within-RSN coupling, we observed differences in SC-FC within the VAN (*F* = 6.085, *η^2^* = 0.033, *p* = 0.003). Compared to HC, within-VAN coupling was lower in CP (*p*_corr_ = 0.012) and CI (*p*_corr_ = 0.006).

Concerning between-RSN coupling, we observed differences in SC-FC in connections between the DAN and the rest of the brain (*F* = 6.037, *η*^2^ = 0.033, *p* = 0.003) and in connections from the SMN to the rest of the brain (*F* = 8.065, *η*^2^ = 0.043, *p* < 0.001). Coupling in DAN-brain connections was higher in CI than in CP (*p*_corr_ = 0.006) and HC (*p*_corr_ = 0.003). Coupling in SMN-brain connections was higher in CI than in HC (*p*_corr_ = 0.003) and CP (*p*_corr_ = 0.024).

Post-hoc analyses of connections between DAN and SMN, respectively, to all other RSNs, showed no significant group differences (*p* > 0.003).

### Variability of SC-FC

Table 3 shows group differences in dSC-FC. Whole-brain dSC-FC was similar between HC, CP, and CI (*p* = 0.758).

**Table 3.** Group comparisons of the variability of structure-function coupling.

	HC (*n* = 95)	CP (*n* = 197)	CI (*n* = 69)	*F*	*η* ^2^	*p*
**(1) Whole-brain coupling variability (α = 0.025)**						
Whole-brain	0.024 ± 0.006	0.023 ± 0.007	0.024 ± 0.007	0.278	0.002	0.758
**(2) Coupling variability within networks (*α* = 0.0035*)***						
Default-mode network	0.052 ± 0.013	0.051 ± 0.012	0.051 ± 0.014	0.170	0.001	0.844
Frontoparietal network	0.080 ± 0.023	0.080 ± 0.020	0.080 ± 0.021	0.020	0.000	0.980
Dorsal attention network	0.073 ± 0.017	0.069 ± 0.018	0.075 ± 0.022	2.561	0.014	0.079
Ventral attention network	0.071 ± 0.018	0.075 ± 0.020	0.074 ± 0.022	0.959	0.005	0.384
Somatomotor network	0.051 ± 0.013	0.052 ± 0.014	0.054 ± 0.017	0.769	0.004	0.464
Visual network	0.041 ± 0.011	0.044 ± 0.013	0.053 ± 0.017	15.373	0.080	<0.001*^a,b^
Deep gray matter	0.087 ± 0.023	0.091 ± 0.027	0.102 ± 0.025	6.908	0.037	0.001*^a,b^
**(3) Coupling variability between networks – rest of brain (*α* = 0.0035)**						
Default-mode network	0.039 ± 0.007	0.038 ± 0.010	0.037 ± .011	0.572	0.003	0.565
Frontoparietal network	0.041 ± 0.010	0.040 ± 0.010	0.041 ± .010	0.179	0.001	0.836
Dorsal attention network	0.035 ± 0.009	0.035 ± 0.008	0.037 ± .010	1.830	0.010	0.162
Ventral attention network	0.039 ± 0.010	0.039 ± 0.010	0.038 ± .010	0.487	0.003	0.615
Somatomotor network	0.042 ± 0.011	0.041 ± 0.011	0.042 ± .011	0.661	0.004	0.517
Visual network	0.056 ± 0.016	0.054 ± 0.015	0.059 ± .019	2.751	0.015	0.065
Deep gray matter	0.047 ± 0.012	0.046 ± 0.013	0.048 ± .013	0.568	0.003	0.567

HC: healthy controls; CP: cognitively preserved people with multiple sclerosis; CI: cognitively impaired people with multiple sclerosis.

Displayed are the mean ± standard deviation. All values are corrected for age, sex, and education level (binarized).

^a^ Significant difference between CP and CI.

^b^ Significant difference between HC and CI.

*Significant group difference at the alpha level as reported in the subheader.

Significant group differences in network-level dSC-FC are shown in Figure 3. Differences in dSC-FC were observed within the VN (*F* = 15.373, *η*^2^ = 0.080, *p* < 0.001) and DGM (*F* = 6.908, *η*^2^ = 0.037, *p* = 0.001). Within-VN dSC-FC was higher in CI than in HC (*p*_corr_ = 0.003) and CP (*p*_corr_ = 0.003). Within-DGM dSC-FC was also higher in CI than in HC (*p*_corr_ = 0.003) and CP (*p*_corr_ = 0.006). No other group differences in dSC-FC were observed.

### Logistic regression of cognitive status

To explore the explanatory power of coupling values, we applied logistic regression (*R*^2^(Nagelkerke) = 0.421; χ*^2^* (df) = 89.822(5), *p* < 0.001), which significantly predicted CP/CI status. Beyond demographics (age removed due to multicollinearity) and measures of structural damage, only SC-FC in DAN-brain connections significantly improved the prediction model for cognitive status (*β* = 0.069, *SE* = 0.031, *Wald* = 4.999, *p* = 0.025). The final model (Table 4) yielded 82.6% accuracy, correctly classifying 93.9% of CP and 50.7% of CI (*F*1 *=* 0.630). The bootstrap analysis (Table 5) demonstrated that beyond structural damage, between-DAN SC-FC indeed significantly predicted CI status in pwMS (*β* = 0.069, bootstrap bias = 0.008, *SE* = 0.036, 95% CI [0.011, 0.156], *p* = 0.041).

**Table 4.** Binomial logistic regression model to predict cognitive status (preserved vs. impaired) in people with MS.

Block		*β*	*SE*	*Wald*	*p*	OR [95% CI]
1	Sex (female vs. male)	1.003	0.362	7.682	0.006*	2.727 [1.342–5.543]
	Education (years)	−0.167	0.103	2.666	0.103	0.846 [0.692–1.034]
2	Normalized GM volume (dl)	−0.142	0.043	11.144	<0.001	0.867 [0.798–0.943]
	Normalized lesion volume (ml)	0.047	0.013	13.200	<0.001	1.049 [1.022–1.076]
3	SC-FC between DAN and rest of the brain	0.069	0.031	4.999	0.025*	1.072 [1.009–1.139]

*SE*: standard error; OR: odds ratio; 95% CI: 95% confidence interval; GM: grey matter; SC-FC: structure connectivity-function coupling; DAN: dorsal attention network; MS: multiple sclerosis.

*Significant effect (*α* = 0.05).

**Table 5.** Bootstrap for binomial logistic regression model to predict cognitive status (preserved vs. impaired) in people with MS.

Block		*Β* [95% CI]	Bias	*SE*	*p*
1	Sex (female vs. male)	1.003 [0.315 to −1.798]	0.036	0.377	0.007*
	Education (years)	−0.167 [−0.404 to −0.034]	−0.019	0.109	0.108
2	Normalized GM volume (dl)	−0.142 [−0.243 to −0.031]	−0.002	0.054	0.007*
	Normalized lesion volume (ml)	0.047 [0.025–0.084]	0.004	0.015	<0.001*
3	SC-FC between DAN and rest of the brain	0.069 [0.011–0.156]	0.008	0.036	0.041*

*SE*: standard error; OR: odds ratio; 95% CI: 95% confidence interval; GM: grey matter; SC-FC: structure connectivity-function coupling; DAN: dorsal attention network; MS: multiple sclerosis.

*Significant effect (*α* = 0.05).

### Sensitivity analyses of SC and FC strength

Whole-brain SC strength was lower in CI compared to CP and HC (*p* < 0.001), and in all RSNs but the FPN (*p* = 0.051). SC strength in connections between RSNs and the rest of the brain was consistently lower in CI versus CP and HC (Supplemental Table S2). Within-SMN and within-VN FC strength was lower in CI compared to CP and HC (Supplemental Table S3).

### SC-FC: Disability status

No group differences were observed based on physical disability (Supplemental Table S4).

### Validation analyses

When repeating analyses including the MCI group, we found group differences in SMN-brain SC-FC, within-VN dSC-FC, and within-DGM dSC-FC (Supplemental Table S5). Regarding GM volume, we found that after correction for multiple comparisons, normalized GM volume significantly contributed only to the model for within-DMN dSC-FC (*F* = 8.760, *η*^2^ = 0.024*, p* = 0.003; Supplemental Table S6).

For DWI, we observed higher mean framewise displacement in PwMS than HC (HC: 0.300 ± 0.100, PwMS: 0.338 ± 0.154, Mann-Whitney *U* = 14,434, *z* = 2.060, *p* = 0.039), but no differences on fMRI (HC: 0.081 ± 0.046, PwMS: 0.086 ± 0.051, Mann-Whitney *U* = 12,862, *z* = 0.260, *p* = 0.795). The group effect in the model for between-DAN SC-FC remained significant, but no longer survived multiple testing correction after adding motion to the model (*F* = 5.379, *η^2^* = 0.029, *p* = 0.005; Supplemental Table S7). For SC strength, motion did not alter the group effects (Supplemental Table S8).

### Cognitive abilities

In PwMS, higher DAN-brain SC-FC significantly predicted worse information processing speed (*β*_std_ = −0.155, *p* = 0.003), recall of verbal information (*β*_std_ = −0.105, *p* = 0.049), and behavioral inhibition (*β*_std_ = −0.108, *p* = 0.051). SC-FC in connections between SMN and the rest of the brain did not predict any cognitive ability score.

Within-VN dSC-FC predicted all cognitive abilities beyond demographics, namely mental flexibility (*β*_std_ *=* −0.173, *p* *=* 0.001), recall of verbal information (*β*_std_ *=* −0.134, *p* *=* 0.012), information processing speed (*β*_std_ *=* −0.235, *p* *<* 0.001), recall of spatial information (*β*_std_ *=* −0.131, *p* *=* 0.016), working memory (*β*_std_ *=* −0.148, *p* *=* 0.005), and behavioral inhibition (*β*_std_ *=* −0.135, *p* *=* 0.014).

Within-DGM dSC-FC predicted mental flexibility (*β*_std_ *=* −0.164, *p* *=* 0.002), information processing speed (*β*_std_ *=* −0.201, *p* *<* 0.001), working memory (*β*_std_ *=* −0.114, *p* *=* 0.033), and behavioral inhibition (*β*_std_ *=* −0.187, *p* *<* 0.001).

## Discussion

Here, we investigated how cognitive impairment in MS is related to the interaction between the brain's structural and functional networks. Generally, higher coupling (SC-FC) and higher coupling variability (dSC-FC) were related to worse cognition in PwMS. While no whole-brain effects were found, network-level group differences were observed between HC, CP, and CI. SC-FC was higher in CI compared to CP and HC for connections between the DAN and SMN and the rest of the brain. Conversely, the time-varying variability of coupling (dSC-FC) was higher within the VN and the DGM in CI compared to HC and CP, highlighting that static and dynamic SC-FC differentially relate to CI.

Congruent with our hypotheses, SC-FC in connections between the DAN and the rest of the brain was higher in CI compared to HC and CP. Possibly, higher constraints of structure on function compromise the spatial multiformity of FC patterns, ensuring more overlap between structure and function.^4^ Since the DAN regulates top-down goal-directed selection of stimuli,^11^ reduced multiformity may hinder the cross-network coordination necessary to orient attention toward task-relevant stimuli. Hindrance of neuronal signaling by structural damage across tracts might explain why higher between-DAN SC-FC predicts slower information processing speed. Important to note is that limbic subcortical structures and cerebellum were excluded, and thus some connections known to be important for cognitive processes are not among the DAN-brain connections described here. Further, after correcting for motion on the diffusion images, this effect did not survive multiple testing correction.

Interestingly, *within*-DAN coupling *variability* (dSC-FC) was related to physical disability. Since the DAN is also involved in motor inhibition,^28^ more variable connectivity may reflect unstable connectivity, possibly hindering motor functions. Together, this suggests that dSC-FC is distinct from SC-FC and related to different types of symptoms.

Surprisingly, the SMN also showed higher coupling in CI as compared to CP and HC. Although typically linked to physical disability,^29^ the SMN also directs movements through top-down cognitive control,^30^ implying a more prominent role for the SMN in cognition than perhaps previously assumed. Again, higher coupling may restrict the multiformity of FC between the SMN and other networks. However, coupling across these tracts predicted neither cognitive status nor cognitive abilities, and was unrelated to disability status. Prior studies in early MS demonstrated lower SC-FC *within* the SMN,^6,31^ but have not examined tracts between the SMN and non-motor areas.

Contrary to our hypotheses, coupling variability (dSC-FC) was higher in CI within the VN and the DGM. Higher within-VN dSC-FC predicted worse scores on all cognitive abilities. In line with this, altered VN dynamics have previously been linked to MS-related cognitive impairment; CI PwMS show reduced distinction between internally oriented (DMN) and externally oriented (visual) states.^13^ Interestingly, evidence in early MS showed *decreased* coupling within the VN alongside limited cognitive impairment.^6^ Perhaps, after initial within-VN SC-FC abnormalities, coupling becomes unstable in later disease stages, expressed as higher coupling variability. Similar differences in coupling variability were observed within the DGM and predicted various cognitive abilities. Since DGM structures are at the core of cognitive functions such as information processing and memory,^32,33^ unstable structure-function interactions in connections between DGM regions may indeed critically impair many different cognitive abilities.

Unexpectedly, the DMN and FPN did not show any group differences between HC, CP, and CI. Potentially, while large hub networks like the DMN do show functional changes in CI, the wide range of structural connections might still ensure normal coupling values overall.^27^ However, the DAN has more specific and sparse connections,^8^ thus even minor structural alterations may limit functional multiformity, inducing cognitive impairment. Further, the VAN showed *lower* within-network coupling in *both MS groups* compared to the HC. The VAN directs attention to stimuli that unconsciously emerge into awareness,^11^ and subsequently activates the DMN (internal stimuli) or the FPN (external stimuli).^34^ Evidence suggests that a more central position of the VAN in the brain's functional network may protect against cognitive decline.^35^ Thus, the VAN possibly helps maintain normal network communication with the rest of the brain in the face of structural damage. Longitudinal studies are needed to clarify the trajectory of these network changes over time and whether they are associated with better or worse cognition.

Some limitations should be taken into account when interpreting our findings. First, despite being known to be important for cognition, several limbic regions and the cerebellum were excluded due to distortion on fMRI. Therefore, the structural and functional networks were imperfectly represented here, and the limbic network could not be evaluated. Second, congruent with previous studies,^6,7^ we calculated SC-FC only for region pairs with a direct structural connection (about 10% of all theoretically possible connections in the brain) and did not include indirect connections, for which alternative approaches are currently being developed.^36^ Further, our diffusion protocol was not optimally sensitive to subtle microstructural changes, yielding higher uncertainty in regions of complex fiber architecture and potentially leading to conservative estimates of connectivity alterations. Moreover, given the difference in DWI-derived mean framewise displacement, our findings are subject to motion-induced bias. Additionally, this article did not inspect the impact of other factors like anxiety and depression, which should be evaluated in subsequent work. Finally, we found small effect sizes, and therefore these results should be interpreted with some caution. As such, while (d)SC-FC provides a new mechanistic way of looking at the brain, this measure might not suffice as a marker of CI in MS. Still, the proposed conceptual framework may aid in a better understanding of the mechanisms underlying MS-related cognitive impairment.

To conclude, this study found higher SC-FC in connections between specific networks (DAN and SMN) and the rest of the brain in CI PwMS, which implies that structural damage limits the functional multiformity of the brain. This constraint possibly constitutes one of multiple mechanisms underlying MS-related cognitive impairment. Further, the variability of coupling appears distinctly associated with MS-related cognitive impairment. Longitudinal work is needed to better understand the interaction between structure and function across the disease trajectory.

## Supplemental Material

sj-docx-1-mso-10.1177_20552173261448282 - Supplemental material for The constrained brain in multiple sclerosis: Cognitive impairment is related to network-specific coupling of structural and functional connectivity Supplemental material, sj-docx-1-mso-10.1177_20552173261448282 for The constrained brain in multiple sclerosis: Cognitive impairment is related to network-specific coupling of structural and functional connectivity by Marloes DA Bet, Maureen van Dam, Tommy AA Broeders, Aurélie Ruet, Frederik Barkhof, Eva MM Strijbis, Linda Douw, Ismail Koubiyr, Hanneke E Hulst and Menno M Schoonheim in Multiple Sclerosis Journal – Experimental, Translational and Clinical

sj-doc-2-mso-10.1177_20552173261448282 - Supplemental material for The constrained brain in multiple sclerosis: Cognitive impairment is related to network-specific coupling of structural and functional connectivity Supplemental material, sj-doc-2-mso-10.1177_20552173261448282 for The constrained brain in multiple sclerosis: Cognitive impairment is related to network-specific coupling of structural and functional connectivity by Marloes DA Bet, Maureen van Dam, Tommy AA Broeders, Aurélie Ruet, Frederik Barkhof, Eva MM Strijbis, Linda Douw, Ismail Koubiyr, Hanneke E Hulst and Menno M Schoonheim in Multiple Sclerosis Journal – Experimental, Translational and Clinical
