# Supplementary material for: The constrained brain in multiple sclerosis: Cognitive impairment is related to network-specific coupling of structural and functional connectivity
Source: Mult Scler J Exp Transl Clin. 2026 Jun 5;12(2):20552173261448282. doi: 10.1177/20552173261448282 (PMC13241616; doi:10.1177/20552173261448282)
Supplement: sj-docx-1-mso-10.1177_20552173261448282 - Supplemental material for The constrained brain in multiple sclerosis: Cognitive impairment is related to network-specific coupling of structural and functional connectivity [file sj-docx-1-mso-10.1177_20552173261448282.docx]

Supplemental Material

# Supplemental Methods

The sample described in this work, or subsets thereof, have previously been investigated in the context of cognitive impairment and beyond. From the diffusion data in this sample, measures of (micro)structural integrity, such as fractional anisotropy, were derived.^1-10^ In some instances, tractography was applied to diffusion data to construct structural networks on which to perform further analyses.^11-14^ Concerning function, both static^1, 2, 15-23^ and dynamic^20, 24-27^ measures of functional connectivity have been investigated. Structure-function coupling has previously been described in an overlapping subset of this sample using MEG.^14^ The current approach to calculating structure-function coupling using MRI-derived structural and functional networks, focusing on connections within and between individual resting-state networks, is unique to this study.

**Table S1**. Excluded regions.

| **Index** | **Region name** | **RSN** | **Anatomical location** |
| --- | --- | --- | --- |
| 27 | Left_A10l, lateral area10 | DMN | Middle Frontal Gyrus |
| 28 | Right_A10l, lateral area10 | FPN | Middle Frontal Gyrus |
| 45 | Left_A11l, lateral area 11 | LN | Orbital Gyrus |
| 46 | Right_A11l, lateral area 11 | LN | Orbital Gyrus |
| 47 | Left_A11m, medial area 11 | LN | Orbital Gyrus |
| 48 | Right_A11m, medial area 11 | LN | Orbital Gyrus |
| 49 | Left_A13, area 13 | LN | Orbital Gyrus |
| 50 | Right_A13, area 13 | LN | Orbital Gyrus |
| 81 | Left_A21c, caudal area 21 | DMN | Middle Temporal Gyrus |
| 82 | Right_A21c, caudal area 21 | DMN | Middle Temporal Gyrus |
| 89 | Left_A20iv, intermediate ventral area 20 | LN | Inferior Temporal Gyrus |
| 90 | Right_A20iv, intermediate ventral area 20 | LN | Inferior Temporal Gyrus |
| 91 | Left_A37elv, extreme lateroventral area37 | DAN | Inferior Temporal Gyrus |
| 92 | Right_A37elv, extreme lateroventral area37 | DAN | Inferior Temporal Gyrus |
| 93 | Left_A20r, rostral area 20 | LN | Inferior Temporal Gyrus |
| 94 | Right_A20r, rostral area 20 | LN | Inferior Temporal Gyrus |
| 95 | Left_A20il, intermediate lateral area 20 | DMN | Inferior Temporal Gyrus |
| 96 | Right_A20il, intermediate lateral area 20 | LN | Inferior Temporal Gyrus |
| 99 | Left_A20cl, caudolateral of area 20 | FPN | Inferior Temporal Gyrus |
| 100 | Right_A20cl, caudolateral of area 20 | FPN | Inferior Temporal Gyrus |
| 101 | Left_A20cv, caudoventral of area 20 | LN | Inferior Temporal Gyrus |
| 102 | Right_A20cv, caudoventral of area 20 | LN | Inferior Temporal Gyrus |
| 109 | Left_A35/36r, rostral area 35/36 | LN | Parahippocampal Gyrus |
| 110 | Right_A35/36r, rostral area 35/36 | LN | Parahippocampal Gyrus |
| 115 | Left_A28/34, area 28/34 (EC, entorhinal cortex) | LN | Parahippocampal Gyrus |
| 116 | Right_A28/34, area 28/34 (EC, entorhinal cortex) | LN | Parahippocampal Gyrus |
| 117 | Left_TI, area TI (temporal agranular insular cortex) | LN | Parahippocampal Gyrus |
| 118 | Right_TI, area TI (temporal agranular insular cortex) | LN | Parahippocampal Gyrus |
| 217 | left nucleus accumbens (derived from FSL-FIRST) | DGM | Deep Gray Matter |
| 224 | right nucleus accumbens (derived from FSL-FIRST) | DGM | Deep Gray Matter |

The atlas regions reported here were excluded from analyses because they showed <30% voxel coverage in ≥10% of included participants. Consequently, the limbic network was excluded from all analyses in its entirety. Region indices were based on a composite atlas including 224 regions. Region indices up until 210 are based on the cortical regions of the Brainnetome atlas; region indices higher than 210 are derived from FSL-FIRST. *Abbreviations: DMN=Default-Mode Network; FPN=Frontoparietal Network; LN=Limbic Network; DAN=Dorsal Attention Network; DGM=Deep Gray Matter.*

**Table S2.** Group comparisons of structural network strength.

|  | | **HC** (*n*=95) | **CP** (*n*=197) | **CI** (*n*=69) | ***F*** | ***η^2^*** | ***p*** |
| --- | --- | --- | --- | --- | --- | --- | --- |
| ***1) Whole-brain structural network strength (α=0.025)*** | | | | | | |  |
|  | Whole-brain | 1.629±0.237 | 1.572±0.219 | 1.452±0.239 | 11.697 | 0.062 | ***<0.001*^a,b,c^*** |
| ***2) Structural network strength within networks (α=0.0035)*** | | | | | | |  |
|  | Default-Mode Network | 0.634±0.100 | 0.620±0.100 | 0.588±0.092 | 4.557 | 0.025 | 0.011 |
|  | Frontoparietal Network | 0.611±0.114 | 0.604±0.101 | 0.571±0.116 | 2.995 | 0.017 | 0.051 |
|  | Dorsal Attention Network | 0.501±0.079 | 0.493±0.078 | 0.465±0.083 | 4.200 | 0.023 | 0.016 |
|  | Ventral Attention Network | 0.235±0.039 | 0.222±0.037 | 0.203±0.042 | 12.963 | 0.069 | ***<0.001*^a,b,c^*** |
|  | Somatomotor Network | 0.870±0.123 | 0.853±0.117 | 0.797±0.130 | 7.244 | 0.040 | ***<0.001*^b,c^*** |
|  | Visual Network | 1.541±0.229 | 1.516±0.228 | 1.401±0.247 | 7.891 | 0.043 | ***<0.001*^b,c^*** |
|  | Deep Gray Matter | 0.678±0.139 | 0.553±0.131 | 0.416±0.153 | 68.992 | 0.282 | ***<0.001*^a,b,c^*** |
| ***3) Structural network strength between networks – rest of brain (α=0.0035)*** | | | | | | |  |
|  | Default-Mode Network | 10.143±0.54 | 9.623±0.751 | 8.730±1.064 | 64.013 | 0.267 | ***<0.001*^a,b,c^*** |
|  | Frontoparietal Network | 5.752±0.36 | 5.342±0.545 | 4.670±0.828 | 67.606 | 0.278 | ***<0.001*^a,b,c^*** |
|  | Dorsal Attention Network | 7.029±0.48 | 6.453±0.644 | 5.665±0.937 | 77.021 | 0.304 | ***<0.001*^a,b,c^*** |
|  | Ventral Attention Network | 6.477±0.37 | 6.108±0.496 | 5.480±0.679 | 72.181 | 0.291 | ***<0.001*^a,b,c^*** |
|  | Somatomotor Network | 8.270±0.48 | 7.726±0.688 | 6.949±0.954 | 66.762 | 0.275 | ***<0.001*^a,b,c^*** |
|  | Visual Network | 4.519±0.38 | 4.119±0.462 | 3.683±0.608 | 58.642 | 0.250 | ***<0.001*^a,b,c^*** |
|  | Deep Gray Matter | 6.571±0.32 | 6.109±0.511 | 5.429±0.728 | 92.282 | 0.344 | ***<0.001*^a,b,c^*** |

Displayed are the mean±standard deviation. All values are corrected for age, sex, and educational level (binarized). Reported *p*-values are not corrected for multiple testing. *^a^*Significant difference between HC and CP. *^b^*Significant difference between CP and CI. *^c^*Significant difference between HC and CI. *Abbreviations: CP=Cognitively Preserved People with Multiple Sclerosis; CI=Cognitively Impaired People with Multiple Sclerosis.*

**Table S3.** Group comparisons of functional network strength.

|  | | **HC** (*n*=95) | **CP** (*n*=197) | **CI** (*n*=69) | ***F*** | ***η^2^*** | ***p*** |
| --- | --- | --- | --- | --- | --- | --- | --- |
| ***1) Whole-brain functional strength (α=0.025)*** | | | | | | |  |
|  | Whole-brain | 61.609±16.896 | 57.739±14.925 | 56.252±14.452 | 3.139 | 0.017 | 0.045 |
| ***2) Functional network strength within networks (α=0.0035)*** | | | | | | |  |
|  | Default-Mode Network | 16.353±3.365 | 15.804±3.199 | 15.855±3.604 | 0.944 | 0.005 | 0.390 |
|  | Frontoparietal Network | 8.309±1.937 | 8.232±1.761 | 8.359±1.957 | 0.143 | 0.001 | 0.866 |
|  | Dorsal Attention Network | 10.868±3.167 | 10.997±3.385 | 10.068±2.678 | 2.271 | 0.013 | 0.105 |
|  | Ventral Attention Network | 10.855±3.047 | 10.527±2.763 | 10.234±2.710 | 0.996 | 0.006 | 0.370 |
|  | Somatomotor Network | 18.762±6.443 | 16.676±5.759 | 16.066±5.251 | 6.098 | 0.033 | ***0.002*^a,c^*** |
|  | Visual Network | 19.227±5.916 | 17.748±5.860 | 15.389±4.705 | 9.230 | 0.049 | ***<0.001*^a,b,c^*** |
|  | Deep Gray Matter | 4.508±1.260 | 4.170±1.058 | 4.022±1.082 | 4.304 | 0.024 | 0.014 |
| ***3) Functional network strength between networks – rest of brain (α=0.0035)*** | | | | | | |  |
|  | Default-Mode Network – rest of brain | 11.197±3.130 | 10.683±2.698 | 10.637±2.831 | 1.273 | 0.007 | 0.281 |
|  | Frontoparietal Network – rest of brain | 5.627±1.483 | 5.434±1.332 | 5.568±1.403 | 0.716 | 0.004 | 0.489 |
|  | Dorsal Attention Network – rest of brain | 7.610±2.391 | 7.244±2.207 | 6.972±1.909 | 1.859 | 0.010 | 0.157 |
|  | Ventral Attention Network – rest of brain | 6.989±2.154 | 6.541±1.709 | 6.568±1.796 | 2.131 | 0.012 | 0.120 |
|  | Somatomotor Network – rest of brain | 10.364±3.717 | 9.394±3.166 | 9.169±3.023 | 3.854 | 0.021 | 0.022 |
|  | Visual Network – rest of brain | 9.167±3.013 | 8.277±2.809 | 7.817±2.541 | 5.434 | 0.030 | 0.005 |
|  | Deep Gray Matter – rest of brain | 3.127±0.839 | 2.945±0.750 | 2.942±0.830 | 1.883 | 0.010 | 0.154 |

Displayed are the mean±standard deviation. All values are corrected for age, sex, and educational level (binarized). Reported *p*-values are not corrected for multiple testing. *^a^*Significant difference between HC and CP. *^b^*Significant difference between CP and CI. *^c^*Significant difference between HC and CI. *Abbreviations: CP=Cognitively Preserved People with Multiple Sclerosis; CI=Cognitively Impaired People with Multiple Sclerosis.*

**Table S4.** Structure-function coupling compared among high and low levels of disability.

|  | ***EDSS ≤ 4.0***  *(n=198)* | ***EDSS > 4.0***  *(n=66)* | ***F*** | ***η^2^*** | ***p*** |
| --- | --- | --- | --- | --- | --- |
| ***1) Whole-brain coupling (α=0.025)*** |  |  |  |  |  |
| Whole-brain | 0.299±0.043 | 0.294±0.066 | 0.514 | 0.002 | 0.474 |
| ***2) Coupling within networks*** ***(α=0.0035)*** |  |  |  |  |  |
| Default-Mode Network | 0.372±0.068 | 0.357±0.095 | 1.587 | 0.006 | 0.209 |
| Frontoparietal Network | 0.341±0.101 | 0.354±0.126 | 0.615 | 0.002 | 0.434 |
| Dorsal Attention Network | 0.324±0.102 | 0.306±0.118 | 1.198 | 0.005 | 0.275 |
| Ventral Attention Network | 0.353±0.104 | 0.370±0.137 | 1.011 | 0.004 | 0.316 |
| Somatomotor Network | 0.368±0.083 | 0.348±0.101 | 2.144 | 0.008 | 0.144 |
| Visual Network | 0.283±0.087 | 0.290±0.092 | 0.313 | 0.001 | 0.577 |
| Deep Gray Matter | 0.149±0.129 | 0.174±0.159 | 1.365 | 0.005 | 0.244 |
| ***3) Coupling between networks – rest of brain (α=0.0035)*** | | | | | |
| Default-Mode Network – rest of brain | 0.254±0.052 | 0.244±0.064 | 1.324 | 0.005 | 0.251 |
| Frontoparietal Network – rest of brain | 0.202±0.061 | 0.201±0.072 | 0.016 | 0.000 | 0.899 |
| Dorsal Attention Network – rest of brain | 0.241±0.051 | 0.239±0.071 | 0.072 | 0.000 | 0.788 |
| Ventral Attention Network – rest of brain | 0.251±0.053 | 0.248±0.065 | 0.149 | 0.001 | 0.700 |
| Somatomotor Network – rest of brain | 0.260±0.058 | 0.258±0.073 | 0.019 | 0.000 | 0.891 |
| Visual Network – rest of brain | 0.265±0.079 | 0.250±0.087 | 1.515 | 0.006 | 0.220 |
| Deep Gray Matter – rest of brain | 0.143±0.077 | 0.140±0.061 | 0.092 | 0.000 | 0.762 |
| ***1) Whole-brain coupling variability (α=0.025)*** | | | | | |
| Whole-brain | 0.023±0.006 | 0.024±0.007 | 0.230 | 0.001 | 0.632 |
| ***2) Coupling variability within networks*** ***(α=0.0035)*** | | |  |  |  |
| Default-Mode Network | 0.051±0.013 | 0.050±0.013 | 0.492 | 0.002 | 0.484 |
| Frontoparietal Network | 0.080±0.020 | 0.081±0.022 | 0.240 | 0.001 | 0.624 |
| Dorsal Attention Network | 0.069±0.017 | 0.077±0.023 | 7.287 | 0.027 | 0.007 |
| Ventral Attention Network | 0.073±0.019 | 0.079±0.025 | 3.839 | 0.015 | 0.051 |
| Somatomotor Network | 0.052±0.014 | 0.053±0.018 | 0.186 | 0.001 | 0.666 |
| Visual Network | 0.046±0.014 | 0.048±0.015 | 0.934 | 0.004 | 0.335 |
| Deep Gray Matter | 0.095±0.028 | 0.093±0.026 | 0.193 | 0.001 | 0.660 |
| ***3) Coupling variability between networks – rest of brain (α=0.0035)*** | | |  |  |  |
| Default-Mode Network – rest of brain | 0.038±0.010 | 0.038±0.012 | 0.075 | 0.000 | 0.784 |
| Frontoparietal Network – rest of brain | 0.040±0.009 | 0.041±0.012 | 0.501 | 0.002 | 0.480 |
| Dorsal Attention Network – rest of brain | 0.035±0.009 | 0.036±0.009 | 0.513 | 0.002 | 0.474 |
| Ventral Attention Network – rest of brain | 0.038±0.010 | 0.039±0.010 | 0.030 | 0.000 | 0.863 |
| Somatomotor Network – rest of brain | 0.042±0.011 | 0.040±0.010 | 1.325 | 0.005 | 0.251 |
| Visual Network – rest of brain | 0.054±0.015 | 0.057±0.019 | 2.152 | 0.008 | 0.144 |
| Deep Gray Matter – rest of brain | 0.047±0.013 | 0.045±0.013 | 0.749 | 0.003 | 0.387 |

Displayed are the mean±standard deviation. All values are corrected for age, sex, and educational level (binarized). *Abbreviations: EDSS=Expanded Disability Status Scale; SC-FC=Structure-Function Coupling; RSN=Resting-State Network; dSC-FC=Dynamic Variability of Structure-Function Coupling.*

**Table S5.** Structure-function coupling and its variability compared among HC, CP, MCI and CI.

|  | | **HC**  (*n*=95) | **CP**  (*n*=197) | | **MCI**  *(n=58)* | | **CI**  (*n*=69) | ***F*** | | ***η^2^*** | ***p*** |
| --- | --- | --- | --- | --- | --- | --- | --- | --- | --- | --- | --- |
| ***1) Whole-brain coupling (α=0.025)*** | | | | | |  | | | | | |
|  | Whole-brain | 0.289±0.031 | 0.295±0.041 | | 0.301±0.034 | | 0.304±0.068 | 1.962 | | 0.014 | 0.119 |
| ***2) Coupling within networks (α=0.0035)*** | | | | | |  | | | | | |
|  | Default-Mode Network | 0.366±0.060 | 0.369±0.066 | | 0.372±0.060 | | 0.365±0.100 | 0.345 | | 0.003 | 0.793 |
|  | Frontoparietal Network | 0.344±0.099 | 0.340±0.101 | | 0.316±0.093 | | 0.358±0.126 | 1.515 | | 0.011 | 0.210 |
|  | Dorsal Attention Network | 0.293±0.095 | 0.317±0.102 | | 0.326±0.094 | | 0.334±0.119 | 2.141 | | 0.015 | 0.094 |
|  | Ventral Attention Network | 0.400±0.100 | 0.361±0.104 | | 0.371±0.097 | | 0.344±0.133 | 4.348 | | 0.031 | 0.005 |
|  | Somatomotor Network | 0.360±0.073 | 0.367±0.078 | | 0.365±0.073 | | 0.353±0.111 | 0.467 | | 0.003 | 0.706 |
|  | Visual Network | 0.267±0.067 | 0.279±0.082 | | 0.289±0.077 | | 0.298±0.104 | 2.092 | | 0.015 | 0.101 |
|  | Deep Gray Matter | 0.149±0.124 | 0.142±0.125 | | 0.154±0.121 | | 0.184±0.166 | 1.846 | | 0.013 | 0.138 |
| ***3) Coupling between networks – rest of brain (α=0.0035)*** | | | | | |  | | | | | |
|  | Default-Mode Network | 0.247±0.050 | 0.249±0.052 | | 0.246±0.043 | | 0.260±0.065 | 0.876 | | 0.006 | 0.454 |
|  | Frontoparietal Network | 0.199±0.054 | 0.200±0.058 | | 0.209±0.049 | | 0.205±0.080 | 0.613 | | 0.004 | 0.607 |
|  | Dorsal Attention Network | 0.231±0.049 | 0.234±0.051 | | 0.242±0.055 | | 0.259±0.070 | 3.724 | | 0.026 | 0.012 |
|  | Ventral Attention Network | 0.254±0.047 | 0.248±0.052 | | 0.252±0.051 | | 0.256±0.066 | 0.510 | | 0.004 | 0.675 |
|  | Somatomotor Network | 0.238±0.052 | 0.253±0.057 | | 0.263±0.061 | | 0.276±0.072 | 5.788 | | 0.040 | ***<0.001*^a,b,c,e^*** |
|  | Visual Network | 0.234±0.083 | 0.258±0.080 | | 0.277±0.073 | | 0.265±0.086 | 4.064 | | 0.029 | 0.007 |
|  | Deep Gray Matter | 0.128±0.071 | 0.139±0.076 | | 0.149±0.067 | | 0.148±0.066 | 1.551 | | 0.011 | 0.201 |
| ***1) Whole-brain coupling variability (α=0.025)*** | | | | | |  | | | | | |
|  | Whole-brain | 0.024±0.006 | 0.023±0.007 | | 0.025±0.007 | | 0.024±0.007 | 0.743 | | 0.005 | 0.527 |
| ***2) Coupling variability within networks (α=0.0035)*** | | | | | |  | | | | | |
|  | Default-Mode Network | 0.052±0.013 | 0.051±0.012 | | 0.049±0.015 | | 0.051±0.014 | 0.261 | | 0.002 | 0.854 |
|  | Frontoparietal Network | 0.080±0.023 | 0.080±0.020 | | 0.074±0.017 | | 0.080±0.021 | 1.390 | | 0.010 | 0.245 |
|  | Dorsal Attention Network | 0.073±0.017 | 0.069±0.018 | | 0.071±0.018 | | 0.075±0.022 | 1.597 | | 0.011 | 0.189 |
|  | Ventral Attention Network | 0.071±0.018 | 0.075±0.020 | | 0.073±0.023 | | 0.074±0.022 | 0.576 | | 0.004 | 0.631 |
|  | Somatomotor Network | 0.051±0.013 | 0.052±0.014 | | 0.054±0.016 | | 0.054±0.017 | 1.500 | | 0.011 | 0.214 |
|  | Visual Network | 0.041±0.011 | 0.044±0.013 | | 0.046±0.011 | | 0.053±0.017 | 10.718 | | 0.072 | ***<0.001*^b,c,d,e,f^*** |
|  | Deep Gray Matter | 0.087±0.023 | 0.091±0.027 | | 0.094±0.028 | | 0.102±0.025 | 4.622 | | 0.033 | ***0.003*^c,e^*** |
| ***3) Coupling variability between networks – rest of brain (α=0.0035)*** | | | |  | | | | |  | | |
|  | Default-Mode Network | 0.039±0.007 | 0.038±0.010 | | 0.039±0.012 | | 0.037±.011 | 0.550 | | 0.004 | 0.648 |
|  | Frontoparietal Network | 0.041±0.010 | 0.040±0.010 | | 0.040±0.010 | | 0.041±.010 | 0.119 | | 0.001 | 0.949 |
|  | Dorsal Attention Network | 0.035±0.009 | 0.035±0.008 | | 0.036±0.009 | | 0.037±.010 | 1.460 | | 0.011 | 0.225 |
|  | Ventral Attention Network | 0.039±0.010 | 0.039±0.010 | | 0.038±0.010 | | 0.038±.010 | 0.361 | | 0.003 | 0.781 |
|  | Somatomotor Network | 0.042±0.011 | 0.041±0.011 | | 0.041±0.012 | | 0.042±.011 | 0.510 | | 0.004 | 0.676 |
|  | Visual Network | 0.056±0.016 | 0.054±0.015 | | 0.056±0.015 | | 0.059±.019 | 1.858 | | 0.013 | 0.136 |
|  | Deep Gray Matter | 0.047±0.012 | 0.046±0.013 | | 0.044±0.010 | | 0.048±.013 | 1.095 | | 0.008 | 0.351 |

Displayed are the mean±standard deviation. All values are corrected for age, sex, and educational level (binarized). *Significant group difference at the alpha level as reported in the subheader. *^a^*Significant difference between HC and CP. *^b^*Significant difference between HC and CP. *^c^*Significant difference between HC and CI. *^d^*Significant difference between CP and MCI. *^e^*Significant difference between CP and CI. *^f^*Significant difference between MCI and CI. *Abbreviations: HC=Healthy Controls; CP=Cognitively Preserved People with Multiple Sclerosis; MCI=Mildly Cognitively Impaired People with Multiple Sclerosis; CI=Cognitively Impaired People with Multiple Sclerosis.*

**Table S6.** Parameters of models comparing structure-function coupling and its variability among HC, CP, and CI, corrected for grey matter volume.

|  | | ***Group (HC/CP/CI)*** | | | | | | ***Normalized Grey Matter Volume*** | | | |
| --- | --- | --- | --- | --- | --- | --- | --- | --- | --- | --- | --- |
|  | | ***F*** | | ***η^2^*** | | | ***p*** | ***F*** | | ***η^2^*** | ***p*** |
| ***1) Whole-brain coupling (α=0.025)*** | | | | | | | | | | | |
|  | Whole-brain | 1.874 | | 0.010 | | | 0.155 | 0.018 | | <0.001 | 0.893 |
| ***2) Coupling within networks (α=0.0035)*** | | | | | | | | | | | |
|  | Default-Mode Network | **0.162** | | 0.001 | | | 0.851 | 1.160 | | 0.003 | 0.282 |
|  | Frontoparietal Network | 0.637 | | 0.004 | | | 0.529 | <0.001 | | <0.001 | 0.984 |
|  | Dorsal Attention Network | 1.429 | | 0.008 | | | 0.241 | 2.430 | | 0.007 | 0.120 |
|  | Ventral Attention Network | 3.538 | | 0.020 | | | 0.030 | 2.734 | | 0.008 | 0.099 |
|  | Somatomotor Network | 1.093 | | 0.006 | | | 0,336 | 1.413 | | 0.004 | 0.235 |
|  | Visual Network | 1.352 | | 0.008 | | | 0.260 | 0.965 | | 0.003 | 0.327 |
|  | Deep Gray Matter | 1.696 | | 0.009 | | | 0.185 | 0.560 | | 0.002 | 0.455 |
| ***3) Coupling between networks – rest of brain (α=0.0035)*** | | | | | | | | | | | |
|  | Default-Mode Network | 1.980 | | 0.011 | | | 0.140 | 1.920 | | 0.005 | 0.167 |
|  | Frontoparietal Network | 0.856 | | 0.005 | | | 0.426 | 3.220 | | 0.009 | 0.074 |
|  | Dorsal Attention Network | 4.837 | | 0.008 | | | 0.027 | 0.005 | | <0.001 | 0.944 |
|  | Ventral Attention Network | 1.107 | | 0.006 | | | 0.332 | 1.236 | | 0.003 | 0.267 |
|  | Somatomotor Network | 8.302 | | 0.045 | | | ***<0.001*^a,b,c^*** | 0.007 | | <0.001 | 0.933 |
|  | Visual Network | 3.150 | | 0.017 | | | 0.044 | 1.119 | | 0.003 | 0.291 |
|  | Deep Gray Matter | 2.899 | | 0.016 | | | 0.056 | 3.718 | | 0.010 | 0.055 |
| ***1) Whole-brain coupling variability (α=0.025)*** | | |  | | |  | |  | |  |  |
|  | Whole-brain | 0.203 | | 0.001 | | | 0.817 | 0.468 | | 0.001 | 0.494 |
| ***2) Coupling variability within networks (α=0.0035)*** | | | | | | | | | | | |
|  | Default-Mode Network | 0.441 | | 0.002 | | | 0.644 | 8.760 | | 0.024 | ***0.003**** |
|  | Frontoparietal Network | 0.050 | | <0.001 | | | 0.951 | 1.194 | | 0.003 | 0.275 |
|  | Dorsal Attention Network | 2.128 | | 0.012 | | | 0.121 | 4.033 | | 0.011 | ***0.045**** |
|  | Ventral Attention Network | 0.633 | | 0.004 | | | 0.532 | 2.815 | | 0.008 | 0.094 |
|  | Somatomotor Network | 0.740 | | 0.004 | | | 0.478 | 0.051 | | <0.001 | 0.821 |
|  | Visual Network | 7.549 | | 0.041 | | | ***<0.001*^b,c^*** | 6.751 | | 0.019 | ***0.010**** |
|  | Deep Gray Matter | 2.434 | | 0.014 | | | 0.089 | 7.122 | | 0.020 | ***0.008**** |
| ***3) Coupling variability between networks – rest of brain (α=0.0035)*** | | | | |  | | | |  | | |
|  | Default-Mode Network | 0.136 | | 0.001 | | | 0.873 | 1.083 | | 0.003 | 0.299 |
|  | Frontoparietal Network | 0.182 | | 0.001 | | | 0.834 | 0.157 | | <0.001 | 0.692 |
|  | Dorsal Attention Network | 3.025 | | 0.017 | | | 0.050 | 3.312 | | 0.009 | 0.070 |
|  | Ventral Attention Network | 0.029 | | <0.001 | | | 0.972 | 2.390 | | 0.007 | 0.123 |
|  | Somatomotor Network | 1.023 | | 0.006 | | | 0.361 | 3.623 | | 0.010 | 0.058 |
|  | Visual Network | 1.897 | | 0.011 | | | 0.151 | 1.571 | | 0.004 | 0.211 |
|  | Deep Gray Matter | 0.864 | | 0.005 | | | 0.422 | 1.906 | | 0.005 | 0.168 |

All models were corrected for sex, age, and education level (dichotomized). Given correction for multiple comparisons, the group variable was considered a significant predictor of structure-function coupling measures if p<0.025 for whole-brain measures and p<0.0035 for network-level measures. *Significant contribution of column variable to the model predicting the row variable. ^a^Significant difference between HC and CP. ^b^Significant difference between HC and CI. ^c^Significant difference between CP and CI. *Abbreviations. HC=Healthy Controls; CP=Cognitively Preserved People with Multiple Sclerosis; CI=Cognitively Impaired People with Multiple Sclerosis.*

**Table S7.** Parameters of models comparing structure-function coupling and its variability among HC, CP, and CI, corrected for mean framewise displacement on diffusion images.

|  | | | ***Group (HC/CP/CI)*** | | | | | ***Mean frame-wise displacement on DWI*** | | | | | |
| --- | --- | --- | --- | --- | --- | --- | --- | --- | --- | --- | --- | --- | --- |
|  | | | ***F*** | ***η^2^*** | | ***p*** | | ***F*** | | | ***η^2^*** | | ***p*** |
| ***1) Whole-brain coupling (α=0.025)*** | | | | | | | | | | | | | |
|  | Whole-brain | | 1.879 | 0.011 | | 0.154 | | 0.781 | | | 0.002 | | 0.377 |
| ***2) Coupling within networks (α=0.0035)*** | | | | | | | | | | | | | |
|  | Default-Mode Network | | 0.145 | 0.001 | | 0.865 | | 0.907 | | | 0.003 | | 0.341 |
|  | Frontoparietal Network | | 0.510 | 0.003 | | 0.601 | | 3.421 | | | 0.010 | | 0.065 |
|  | Dorsal Attention Network | | 2.946 | 0.016 | | 0.054 | | 0.265 | | | 0.001 | | 0.607 |
|  | Ventral Attention Network | | 5.760 | 0.032 | | ***0.003*^a,b^*** | | 0.238 | | | 0.001 | | 0.626 |
|  | Somatomotor Network | | 0.835 | 0.005 | | 0.435 | | 2.011 | | | 0.006 | | 0.157 |
|  | Visual Network | | 2.691 | 0.015 | | 0.069 | | 0.079 | | | 0.000 | | 0.779 |
|  | Deep Gray Matter | | 2.468 | 0.014 | | 0.086 | | 0.003 | | | 0.000 | | 0.958 |
| ***3) Coupling between networks – rest of brain (α=0.0035)*** | | | | | | | | | | | | | |
|  | Default-Mode Network | | 1.117 | 0.006 | | 0.328 | | 0.064 | | | 0.000 | | 0.800 |
|  | Frontoparietal Network | | 0.071 | 0.000 | | 0.932 | | 2.209 | | | 0.006 | | 0.138 |
|  | Dorsal Attention Network | | 5.379 | 0.029 | | 0.005 | | 1.411 | | | 0.004 | | 0.236 |
|  | Ventral Attention Network | | 0.811 | 0.005 | | 0.445 | | 0.078 | | | 0.000 | | 0.780 |
|  | Somatomotor Network | | 7.276 | 0.039 | | ***<0.001*^a,b,c^*** | | 1.237 | | | 0.003 | | 0.267 |
|  | Visual Network | | 3.789 | 0.021 | | 0.024 | | 0.188 | | | 0.001 | | 0.665 |
|  | Deep Gray Matter | | 1.578 | 0.009 | | 0.208 | | 0.125 | | | 0.000 | | 0.724 |
| ***1) Whole-brain coupling variability (α=0.025)*** | |  | | |  | |  | | |  | |  | |
|  | Whole-brain | | 0.257 | 0.001 | | 0.773 | | 0.099 | | | 0.000 | | 0.754 |
| ***2) Coupling variability within networks (α=0.0035)*** | | | | | | | | | | | | | |
|  | Default-Mode Network | | 0.173 | 0.001 | | 0.841 | | 0.007 | | | 0.00 | | 0.933 |
|  | Frontoparietal Network | | 0.005 | 0.000 | | 0.995 | | 0.546 | | | 0.002 | | 0.460 |
|  | Dorsal Attention Network | | 2.457 | 0.014 | | 0.087 | | 0.679 | | | 0.002 | | 0.410 |
|  | Ventral Attention Network | | 0.954 | 0.005 | | 0.386 | | 0.002 | | | 0.000 | | 0.967 |
|  | Somatomotor Network | | 0.890 | 0.005 | | 0.412 | | 0.568 | | | 0.002 | | 0.452 |
|  | Visual Network | | 14.855 | 0.077 | | ***<0.001*^b,c^*** | | 0.042 | | | 0.000 | | 0.838 |
|  | Deep Gray Matter | | 6.416 | 0.035 | | ***0.002*^b,c^*** | | 0.425 | | | 0.001 | | 0.515 |
| ***3) Coupling variability between networks – rest of brain (α=0.0035)*** | | | | | | | | |  | | | | |
|  | Default-Mode Network | | 0.541 | 0.003 | | 0.583 | | 0.014 | | | 0.000 | | 0.907 |
|  | Frontoparietal Network | | 0.175 | 0.001 | | 0.839 | | 0.079 | | | 0.000 | | 0.779 |
|  | Dorsal Attention Network | | 1.786 | 0.010 | | 0.169 | | 0.007 | | | 0.000 | | 0.934 |
|  | Ventral Attention Network | | 0.346 | 0.002 | | 0.708 | | 1.038 | | | 0.003 | | 0.309 |
|  | Somatomotor Network | | 0.650 | 0.004 | | 0.523 | | 0.020 | | | 0.000 | | 0.888 |
|  | Visual Network | | 2.842 | 0.016 | | 0.060 | | 0.350 | | | 0.001 | | 0.554 |
|  | Deep Gray Matter | | 0.556 | 0.003 | | 0.574 | | 0.775 | | | 0.002 | | 0.379 |

All models were corrected for sex, age, and education level (dichotomized). Given correction for multiple comparisons, the group variable was considered a significant predictor of structure-function coupling measures if p<0.025 for whole-brain measures and p<0.0035 for network-level measures. *Significant contribution of column variable to the model predicting the row variable. ^a^Significant difference between HC and CP. ^b^Significant difference between HC and CI. ^c^Significant difference between CP and CI. *Abbreviations. HC=Healthy Controls; CP=Cognitively Preserved People with Multiple Sclerosis; CI=Cognitively Impaired People with Multiple Sclerosis.*

**Table S8.** Parameters of models comparing structural connectivity strength among HC, CP, and CI, corrected for mean framewise displacement on diffusion images.

|  | | ***Group (HC/CP/CI)*** | | | ***Mean frame-wise displacement on DWI*** | | |
| --- | --- | --- | --- | --- | --- | --- | --- |
|  | | ***F*** | ***η^2^*** | ***p*** | ***F*** | ***η^2^*** | ***p*** |
| ***1) Whole-brain SC strength (α=0.025)*** | | | | | | | |
|  | Whole-brain | 11.198 | 0.060 | ***<0.001*^b,c^*** | 0.140 | 0.000 | 0.709 |
| ***2) SC strength within networks (α=0.0035)*** | | | | | | | |
|  | Default-Mode Network | 4.445 | 0.025 | 0.012 | 0.001 | 0.000 | 0.976 |
|  | Frontoparietal Network | 2.792 | 0.016 | 0.063 | 0.202 | 0.001 | 0.654 |
|  | Dorsal Attention Network | 3.776 | 0.021 | 0.024 | 0.822 | 0.002 | 0.365 |
|  | Ventral Attention Network | 11.841 | 0.063 | ***<0.001*^a,b,c^*** | 1.702 | 0.005 | 0.193 |
|  | Somatomotor Network | 6.641 | 0.036 | ***0.001*^b,c^*** | 0.862 | 0.002 | 0.354 |
|  | Visual Network | 7.805 | 0.043 | ***<0.001*^b,c^*** | 0.021 | 0.000 | 0.886 |
|  | Deep Gray Matter | 65.393 | 0.271 | ***<0.001*^a,b,c^*** | 2.800 | 0.008 | 0.095 |
| ***3) SC strength between networks – rest of brain (α=0.0035)*** | | | | | | | |
|  | Default-Mode Network | 59.932 | 0.255 | ***<0.001*^a,b,c^*** | 5.720 | 0.016 | 0.017 |
|  | Frontoparietal Network | 63.418 | 0.265 | ***<0.001*^a,b,c^*** | 5.598 | 0.016 | 0.019 |
|  | Dorsal Attention Network | 72.488 | 0.292 | ***<0.001*^a,b,c^*** | 5.941 | 0.017 | 0.015 |
|  | Ventral Attention Network | 67.880 | 0.279 | ***<0.001*^a,b,c^*** | 5.309 | 0.015 | 0.022 |
|  | Somatomotor Network | 62.197 | 0.262 | ***<0.001*^a,b,c^*** | 9.654 | 0.027 | ***0.002**** |
|  | Visual Network | 54.855 | 0.238 | ***<0.001*^a,b,c^*** | 5.536 | 0.016 | 0.019 |
|  | Deep Gray Matter | 87.010 | 0.331 | ***<0.001*^a,b,c^*** | 8.699 | 0.024 | ***0.003**** |

All models were corrected for sex, age, and education level (dichotomized). Given correction for multiple comparisons, the group variable was considered a significant predictor of structure-function coupling measures if p<0.025 for whole-brain measures and p<0.0035 for network-level measures. *Significant contribution of column variable to the model predicting the row variable. ^a^Significant difference between HC and CP. ^b^Significant difference between HC and CI. ^c^Significant difference between CP and CI. *Abbreviations. HC=Healthy Controls; CP=Cognitively Preserved People with Multiple Sclerosis; CI=Cognitively Impaired People with Multiple Sclerosis.*

Supplemental References

1. Eijlers AJC, Meijer KA, van Geest Q, Geurts JJG, Schoonheim MM. Determinants of Cognitive Impairment in Patients with Multiple Sclerosis with and without Atrophy. Radiology 2018;288:544-551.

2. Meijer KA, van Geest Q, Eijlers AJC, Geurts JJG, Schoonheim MM, Hulst HE. Is impaired information processing speed a matter of structural or functional damage in MS? Neuroimage Clin 2018;20:844-850.

3. Schoonheim MM, Hulst HE, Brandt RB, et al. Thalamus structure and function determine severity of cognitive impairment in multiple sclerosis. Neurology 2015;84:776-783.

4. Krijnen EA, Broeders TAA, Noteboom S, et al. The cognitive relevance of non-lesional damage to cortical networks in people with multiple sclerosis. J Neurol 2024;271:3203-3214.

5. Eijlers AJC, van Geest Q, Dekker I, et al. Predicting cognitive decline in multiple sclerosis: a 5-year follow-up study. Brain 2018;141:2605-2618.

6. Schoonheim MM, Vigeveno RM, Rueda Lopes FC, et al. Sex-specific extent and severity of white matter damage in multiple sclerosis: implications for cognitive decline. Hum Brain Mapp 2014;35:2348-2358.

7. Daams M, Steenwijk MD, Schoonheim MM, et al. Multi-parametric structural magnetic resonance imaging in relation to cognitive dysfunction in long-standing multiple sclerosis. Mult Scler 2016;22:608-619.

8. Steenwijk MD, Daams M, Pouwels PJ, et al. What explains gray matter atrophy in long-standing multiple sclerosis? Radiology 2014;272:832-842.

9. Bouman PM, van Dam MA, Jonkman LE, et al. Isolated cognitive impairment in people with multiple sclerosis: frequency, MRI patterns and its development over time. J Neurol 2024;271:2159-2168.

10. Krijnen EA, Russo AW, Salim Karam E, et al. Detection of grey matter microstructural substrates of neurodegeneration in multiple sclerosis. Brain Commun 2023;5:fcad153.

11. Meijer KA, Steenwijk MD, Douw L, Schoonheim MM, Geurts JJG. Long-range connections are more severely damaged and relevant for cognition in multiple sclerosis. Brain 2020;143:150-160.

12. Koubiyr I, Krijnen EA, Eijlers AJC, et al. Longitudinal fibre-specific white matter damage predicts cognitive decline in multiple sclerosis. Brain Commun 2024;6:fcae018.

13. Steenwijk MD, Daams M, Pouwels PJ, et al. Unraveling the relationship between regional gray matter atrophy and pathology in connected white matter tracts in long-standing multiple sclerosis. Hum Brain Mapp 2015;36:1796-1807.

14. Kulik SD, Nauta IM, Tewarie P, et al. Structure-function coupling as a correlate and potential biomarker of cognitive impairment in multiple sclerosis. Netw Neurosci 2022;6:339-356.

15. Meijer KA, Eijlers AJC, Douw L, et al. Increased connectivity of hub networks and cognitive impairment in multiple sclerosis. Neurology 2017;88:2107-2114.

16. Eijlers AJ, Meijer KA, Wassenaar TM, et al. Increased default-mode network centrality in cognitively impaired multiple sclerosis patients. Neurology 2017;88:952-960.

17. Strik M, Chard DT, Dekker I, et al. Increased functional sensorimotor network efficiency relates to disability in multiple sclerosis. Mult Scler 2021;27:1364-1373.

18. Huiskamp M, Eijlers AJC, Broeders TAA, et al. Longitudinal Network Changes and Conversion to Cognitive Impairment in Multiple Sclerosis. Neurology 2021;97:e794-e802.

19. Schoonheim MM, Pinter D, Prouskas SE, et al. Disability in multiple sclerosis is related to thalamic connectivity and cortical network atrophy. Mult Scler 2022;28:61-70.

20. Schoonheim MM, Douw L, Broeders TA, Eijlers AJ, Meijer KA, Geurts JJ. The cerebellum and its network: Disrupted static and dynamic functional connectivity patterns and cognitive impairment in multiple sclerosis. Mult Scler 2021;27:2031-2039.

21. Hindriks R, Broeders TAA, Schoonheim MM, et al. Higher-order functional connectivity analysis of resting-state functional magnetic resonance imaging data using multivariate cumulants. Hum Brain Mapp 2024;45:e26663.

22. Meijer KA, Eijlers AJC, Geurts JJG, Schoonheim MM. Staging of cortical and deep grey matter functional connectivity changes in multiple sclerosis. J Neurol Neurosurg Psychiatry 2018;89:205-210.

23. Dekker I, Schoonheim MM, Venkatraghavan V, et al. The sequence of structural, functional and cognitive changes in multiple sclerosis. Neuroimage Clin 2021;29:102550.

24. Broeders TAA, Douw L, Eijlers AJC, et al. A more unstable resting-state functional network in cognitively declining multiple sclerosis. Brain Commun 2022;4:fcac095.

25. Eijlers AJC, Wink AM, Meijer KA, Douw L, Geurts JJG, Schoonheim MM. Reduced Network Dynamics on Functional MRI Signals Cognitive Impairment in Multiple Sclerosis. Radiology 2019;292:449-457.

26. Broeders TAA, van Dam M, Pontillo G, et al. Energy Associated With Dynamic Network Changes in Patients With Multiple Sclerosis and Cognitive Impairment. Neurology 2024;103:e209952.

27. Strik M, Eijlers AJ, Dekker I, et al. Sensorimotor network dynamics predict decline in upper and lower limb function in people with multiple sclerosis. Mult Scler 2022:13524585221125372.
