# Supplementary material for: The constrained brain in multiple sclerosis: Cognitive impairment is related to network-specific coupling of structural and functional connectivity
Source: Mult Scler J Exp Transl Clin. 2026 Jun 5;12(2):20552173261448282. doi: 10.1177/20552173261448282 (PMC13241616; doi:10.1177/20552173261448282)
Supplement: sj-doc-2-mso-10.1177_20552173261448282 - Supplemental material for The constrained brain in multiple sclerosis: Cognitive impairment is related to network-specific coupling of structural and functional connectivity [file sj-doc-2-mso-10.1177_20552173261448282.doc]

**MS Journal Appendix for MRI methodology**

| Hardware | |
| --- | --- |
| Field strength | 3T |
| Manufacturer | GE |
| Model | Signa-HDxt |
| Coil type  (e.g. head, surface) | head coil |
| Number of coil channels | 8 |

| Acquisition sequence | | |
| --- | --- | --- |
| Type  (e.g. FLAIR, DIR, DTI, fMRI) | T1, FLAIR, DTI, fMRI | |
| Acquisition time | T1: ~5 min  FLAIR: ~5 min,  DTI: ~5 min,  fMRI: ~8 min | |
| Orientation | all sagittal | |
| Alignment  (e.g. anterior commissure/poster commissure line) | all anterior/posterior commissure line | |
| Voxel size | T1: 0.94x0.94x1.0 mm  FLAIR: 0.98x0.98x1.2 mm  DTI: 2.0x2.0x2.4 mm  fMRI: 3.3x3.3x3.0 mm | |
| TR | T1: 7.8ms  FLAIR: 8000ms  DTI: 13ms  fMRI: 2200ms | |
| TE | T1: 3.22ms  FLAIR: 125ms  DTI: 91ms  fMRI: 35ms | |
| TI | T1: 450 ms  FLAIR: 2350ms | |
| Flip angle | T1: 12  FLAIR: 90  DTI: 90  fMRI: 20 | |
| NEX | 1 / N/A | |
| Field of view | T1: 162x241  FLAIR: 130x251  DTI: 512x512  fMRI: 211x211 | |
| Matrix size | T1: 172x256x256  FLAIR: 132x256x256  DTI: 256x256x53x35  fMRI: 64x64x42x202 | |
| Parallel imaging | Yes | **No** |
| If used, parallel imaging method:  (e.g. SENSE, GRAPPA) |  | |
| Cardiac gating | Yes | **No** |
| If used, cardiac gating method:  (e.g. PPU or ECG) |  | |
| Contrast enhancement | Yes | **No** |
| If used, provide name of contrast agent, dose and timing of scan post-contrast administration |  | |
| Other parameters: |  | |

| Image analysis methods and outputs | |
| --- | --- |
| ***Lesions*** | |
| Type  (e.g. Gd-enhancing, T2-hyperintense, T1-hypointense) |  |
| Analysis method |  |
| Analysis software |  |
| Output measure  (e.g. count or volume [ml]) |  |
| ***Tissue volumes*** | |
| Type  (e.g. whole brain, grey matter, white matter, spinal cord) |  |
| Analysis method |  |
| Analysis software |  |
| Output measure  (e.g. absolute tissue volume in ml, tissue volume as a fraction of intracranial volume, percentage change in tissue volumes) |  |
| ***Tissue measures (e.g. MTR, DTI, T1-RT, T2-RT, T2*, T2’, ^1^H-MRS, perfusion, Na)*** | |
| Type  (e.g. whole brain, grey matter, white matter, spinal cord, normal-appearing grey matter or white matter) |  |
| Analysis method |  |
| Analysis software |  |
| Output measure |  |
| ***Other MRI measures (e.g. functional MRI)*** | |
| Type  (e.g. whole brain, grey matter, white matter, spinal cord, normal-appearing grey matter or white matter) |  |
| Analysis method |  |
| Analysis software |  |
| Output measure |  |

**Other analysis details:**
